# The heterogeneous sensitivity of pediatric brain tumors to different oncolytic viruses is predicted by unique gene expression profiles

**DOI:** 10.1016/j.omton.2024.200804

**Published:** 2024-04-15

**Authors:** Konstantinos Vazaios, Εftychia Stavrakaki, Lisette B. Vogelezang, Jie Ju, Piotr Waranecki, Dennis S. Metselaar, Michaël H. Meel, Vera Kemp, Bernadette G. van den Hoogen, Rob C. Hoeben, E. Antonio Chiocca, William F. Goins, Andrew Stubbs, Yunlei Li, Marta M. Alonso, Friso G. Calkoen, Esther Hulleman, Jasper van der Lugt, Martine L.M. Lamfers

**Affiliations:** 1 Princess Máxima Center for Pediatric Oncology, Heidelberglaan 25, 3584 CS Utrecht, the Netherlands; 2 Department of Neurosurgery, Brain Tumor Center, Erasmus Medical Center, Dr. Molewaterplein 40, 3015 GD Rotterdam, the Netherlands; 3 Center for Translational Immunology, University Medical Center Utrecht, Heidelberglaan 100, 3584 CX Utrecht, the Netherlands; 4 Department of Pathology and Clinical Bioinformatics, Erasmus Medical Center, Dr. Molewaterplein 40, 3015 GD Rotterdam, the Netherlands; 5 Department of Pediatrics, Wilhelmina Children’s Hospital, University Medical Center Utrecht, Lundlaan 6, 3584 EA Utrecht, the Netherlands; 6 Department of Cell and Chemical Biology, Leiden University Medical Center, Einthovenweg 20, 2333 ZC Leiden, the Netherlands; 7 Department of Viroscience, Erasmus Medical Center, Dr. Molewaterplein 40, 3015 GD Rotterdam, the Netherlands; 8 Department of Neurosurgery, Brigham and Women’s Hospital, Harvard Medical School, 75 Francis Street, Boston, MA 02115, USA; 9 Department of Microbiology & Molecular Genetics, University of Pittsburgh School of Medicine, 450 Technology Dr, Pittsburgh, PA 15219, USA; 10 Program in Solid Tumors, Center for Applied Medical Research (CIMA), Avda. de Pío XII, 55, 31008 Pamplona, Spain; 11 Department of Pediatrics, Clínica Universidad de Navarra, Av. de Pío XII, 36, 31008 Pamplona, Spain; 12 Health Research Institute of Navarra (IDISNA), Av. de Pío XII, 36, 31008 Pamplona, Spain

**Keywords:** MT: Regular Issue, pediatric brain tumors, oncolytic viruses, sensitivity, resistance, Gene Ontology, response prediction, *in vitro*, spheres

## Abstract

Despite decades of research, the prognosis of high-grade pediatric brain tumors (PBTs) remains dismal; however, recent cases of favorable clinical responses were documented in clinical trials using oncolytic viruses (OVs). In the current study, we employed four different species of OVs: adenovirus Delta24-RGD, herpes simplex virus rQNestin34.5v1, reovirus R124, and the non-virulent Newcastle disease virus rNDV-F0-GFP against three entities of PBTs (high-grade gliomas, atypical teratoid/rhabdoid tumors, and ependymomas) to determine their *in vitro* efficacy. These four OVs were screened on 14 patient-derived PBT cell cultures and the degree of oncolysis was assessed using an ATP-based assay. Subsequently, the observed viral efficacies were correlated to whole transcriptome data and Gene Ontology analysis was performed. Although no significant tumor type-specific OV efficacy was observed, the analysis revealed the intrinsic biological processes that associated with OV efficacy. The predictive power of the identified expression profiles was further validated *in vitro* by screening additional PBTs. In summary, our results demonstrate OV susceptibility of multiple patient-derived PBT entities and the ability to predict *in vitro* responses to OVs using unique expression profiles. Such profiles may hold promise for future OV preselection with effective oncolytic potency in a specific tumor, therewith potentially improving OV responses.

## Graphical abstract

**Figure:**
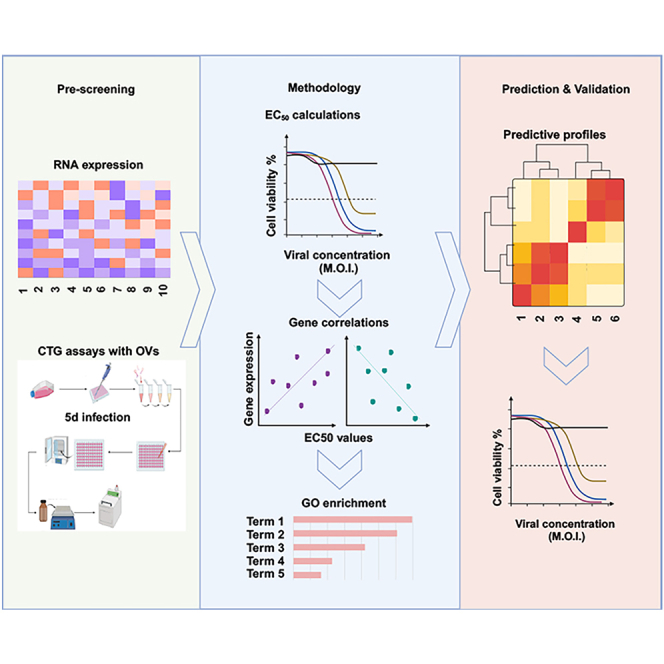

## Teaser

Vazaios and colleagues correlated RNA expression of pediatric brain tumor cells to their *in vitro* sensitivity to four different oncolytic viruses to identify expression profiles for OV sensitivity. Application of the profiles predicted the relative sensitivity/resistance to OV-induced killing in additional tumor cell cultures, providing valuable information for future OV therapies.

## Introduction

Pediatric central nervous system (CNS) tumors are the leading cause of death by disease for age groups 0 to 18.^1^ High-grade gliomas (HGGs) have very poor survival and even in children with gross total resection, the 5-year survival rate is below 20%.^2^ Specifically, the Histone 3 (H3K27M)-altered diffuse midline gliomas (DMGs) carry a median survival of 11 months, largely because they are irresectable.^3^ Atypical teratoid/rhabdoid tumors (AT/RTs) are brain tumors of embryonal origin that account for 1% to 2% of all pediatric CNS tumors in children <3 years old.^4^ Overall, AT/RTs have poor prognosis with 5-year survival rate ≤28%, especially in individuals younger than 3.^5^ Ependymomas (EPNs) are the third most common CNS tumor in children accounting for approximately 10% of cases.^6^ DNA methylation profiling has divided the aforementioned entities into several subgroups each with a preferable anatomic compartment, age of presentation, malignancy grade, and prognosis tightly linked to those groups.^7^^,^^8^^,^^9^^,^^10^ Briefly, according to the Brain classifier version 12.8 (https://www.molecularneuropathology.org/mnp/classifiers/14) from Heidelberg, HGGs are divided into H3 wild type (WT) and IDH WT, H3 G34-mutant, MYCN, RTK1, RTK2, DMG H3K27 and epidermal growth factor receptor (EGFR), DMG H3K27/EZHIP subtypes; AT/RTs are divided into MYC, SHH ,and TYR subtypes; and EPNs are divided into Myxopapillary, Posterior fossa group A (PFA)1a-f, PFA2a-c, PFB1-5, spinal, spinal and MYCN-amplified, Spinal subEPN A and B, supratentorial EPN YAP1-fused, ZFTA fused, ZFTA::RELA fused, and supratentorial subEPN.^10^

The standard treatment for high-grade PBTs consists of a combination of surgical resection, when feasible, followed by chemotherapy and/or radiotherapy. Over the past decades, little improvement in survival has been made compared with other pediatric tumors. Moreover, treatment comes with long-term morbidity, resulting in a significant decline in quality of life.^11^ Therefore, there is an urgent need for alternative and less invasive treatment strategies to reduce the long-term treatment effects, while improving survival rates.

An upcoming alternative option that could help overcome these challenges is the use of oncolytic viruses (OVs). OVs specifically infect tumor cells, replicate, and spread to neighboring uninfected tumor cells, causing tumor cell lysis.^12^^,^^13^^,^^14^ In addition, the oncolytic activity of the OV can trigger a local inflammatory response through the release of pathogen-associated molecular patterns (PAMPS), damage-associated molecular patterns (DAMPS), cytokines, and chemokines. This response, along with the release of tumor-associated antigens (TAAs) from infected tumor cells, subsequently initiates a systemic anti-tumor immune response.^12^^,^^15^^,^^16^^,^^17^ Numerous DNA and RNA virus species, such as adenovirus (AdV), herpes simplex virus (HSV), reovirus (RV), and Newcastle disease virus (NDV), have been tested in preclinical models and have been studied in clinical trials against PBTs with various successes.^18^^,^^19^^,^^20^^,^^21^ More specifically, Delta24-RGD, also known as DNX-2401, is a type 5 human AdV genetically modified with a 24-base pair deletion on its *E1A* gene and an addition of an arginine-glycine-aspartic acid (RGD) motif on its fiber protein.^22^ These modifications enable the virus to interact with α_v_ integrins for attachment and entry and to replicate in cells with a dysfunctional retinoblastoma tumor suppressor (RB) pathway, thereby providing tumor cell specificity.^22^ Promising clinical results were obtained during a recent clinical trial, where DNX-2401 was administered intratumorally in 12 DMG patients, which led to a median overall survival of 17.8 months compared with the historic average of 11 months.^18^ rQNestin34.5v1 is a neurotropic HSV-1 modified to target cells overexpressing Nestin, such as glioblastoma and cancer stem-like cells, and not the surrounding healthy cells, thereby providing tumor selectivity.^23^^,^^24^^,^^25^ R124 is an unmodified human RV type 3 Dearing (T3D), which has been reported to have tropism toward cells with an upregulated Ras/RalGEF/p53 pathway.^19^^,^^26^^,^^27^^,^^28^^,^^29^ This virus displayed highly variable cytotoxicity in a panel of glioblastoma cell lines.^30^ Finally, rNDV-F0-GFP is an avirulent NDV strain La Sota with a mono-basic cleavage site in the fusion protein (F0) for increased virulence, NDVs often, but not consistently, demonstrate a natural preference for cells with deficiencies in the type I interferon (IFN) pathway, as reported for numerous cancer cell types including adult glioblastoma.^21^^,^^31^^,^^32^^,^^33^^,^^34^

Despite an increasing number of viruses being investigated for clinical use, no studies have covered the oncolytic potential of those viruses against an extended number of PBTs. Recent, *in vitro* studies have employed patient-derived monocultures of brain tumor spheres that retain the phenotypic and molecular profile of the original tumor.^35^^,^^36^ Compared with the classical monolayers, tumor cell-derived spheres are able to recapitulate cell-cell, cell-ECM interactions, as well as quiescent and proliferative zones similar to *in vivo* tumors.^36^^,^^37^ Studies on screening of patient-derived tumor spheres and/or spheroids have also demonstrated small molecule drug sensitivities resembling the *in vivo* efficacy or enabled responder identification, stratification to new treatments, or potential combination synergies.^38^^,^^39^^,^^40^^,^^41^ Therefore, applying patient-derived *in vitro* models may provide a suitable platform for preclinical evaluation of OVs and discovery of predictive factors for OV oncolytic efficacy.

In this study, we evaluated the oncolytic potential of four OVs, Delta24-RGD, rQNestin34.5v1, R124, and rNDV-F0-GFP, on a panel of patient-derived tumor sphere cultures covering a range of PBT entities. Our findings highlight the oncolytic potential of these OVs against PBTs and shed light on the molecular factors contributing to the heterogeneous response observed for each virus. By correlating genes and biological processes with the specific sensitivity and resistance to the OVs, we take a first step toward identifying predictive molecular tumor signatures in OV therapy.

## Results

### PBTs express genes related to viral entry for the four OVs

**Figure 1:**
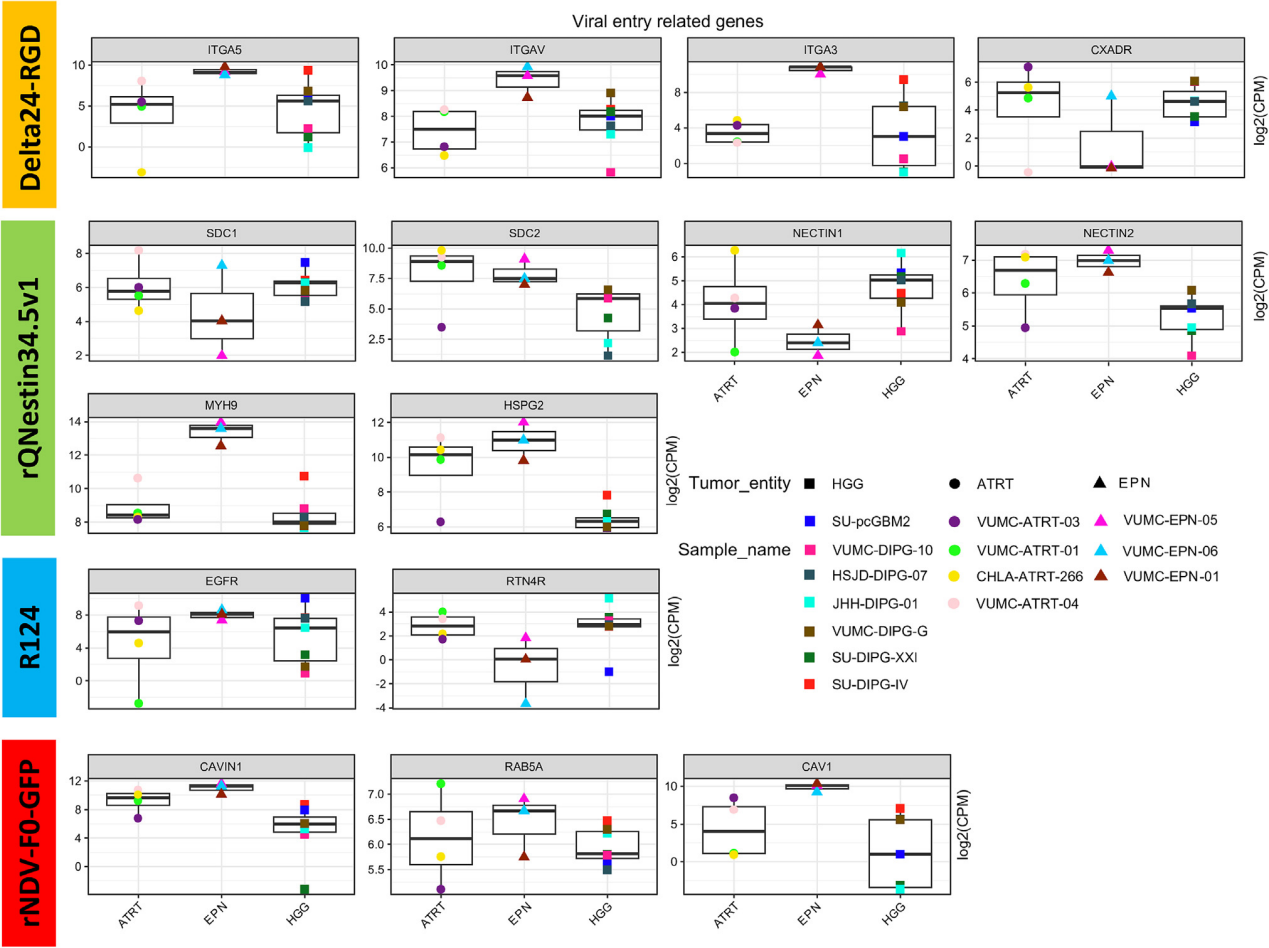
Delta24-RGD, rQNestin34.5v1, R124, and rNDV-F0-GFP entry-related genes in HGG, AT/RT, and EPN Every point represents a unique patient-derived cell culture (*n* = 14) with every sign being a cell culture of specific tumor entity (*n* = 3). Each boxplot corresponds to the median expression of one gene per tumor entity. The y axis represents the transcription levels of the selected genes on a free scale after log2 transformation of the normalized counts per million (CPM), while the x axis represents the tumor entities in which the cell cultures were grouped.

We performed *in silico* analysis of the transcriptome of patient-derived PBT sphere cultures (*n* = 14) belonging to the entities HGG (*n* = 7), AT/RT (*n* = 4), and EPN (*n* = 3). Assessment of RNA levels of genes related to viral cell attachment and entry for the four OVs, as summarized in Table 1, revealed that most viral entry-related genes were expressed by the cell cultures to some degree (Figure 1), with the exception of *HVEM, PILRA*, and *F11R,* which were below detection levels (data not shown). These genes covered entry-related molecules for each of the four OVs, indicating that the selected OVs would be expected to infect patient-derived HGGs, AT/RTs, and EPNs (Figure 1).

**Table 1:** Oncolytic virus entry-related genes mentioned in publications

Oncolytic virus	Viral entry-related genes
Delta24-RGD	IntegrinαVβ5 (*ITGA5*), IntegrinαVβ (*ITGAV*), IntegrinαVβ3 (*ITGA3*), Coxsackie Adenovirus Receptor (*CAR;CXADR*)^42^
rQNestin34.5v1	Herpesvirus Entry Mediator (*HVEM;TNFRSF14*), Syndecan-1 (*SDC1*), Syndecan-2 (*SDC2*), *NECTIN 1, NECTIN 2*, Paired Immunoglobin Like Type 2 Receptor Alpha (*PILRA*), Non-muscle Myosin Heavy Chain IIA (*NMHC-IIA;MYH9*), Perlecan (*HSPG2*)^43^^,^^44^^,^^45^^,^^46^
R124	Junction Adhesion Molecule A (*JAM-A;F11R)*, Epidermal Growth Factor Receptor (*EGFR*), Nogo Receptor (*NgR1;RTN4R*)^26^^,^^30^^,^^47^^,^^48^
rNDV-F0-GFP	Cavin-1 (*CAVIN1*), Ras related protein AB5 (*RAB5*), Caveolin-1 (*CAV1*)^49^^,^^50^

### Oncolytic effect of the OVs in patient-derived PBT cultures

**Figure 2:**
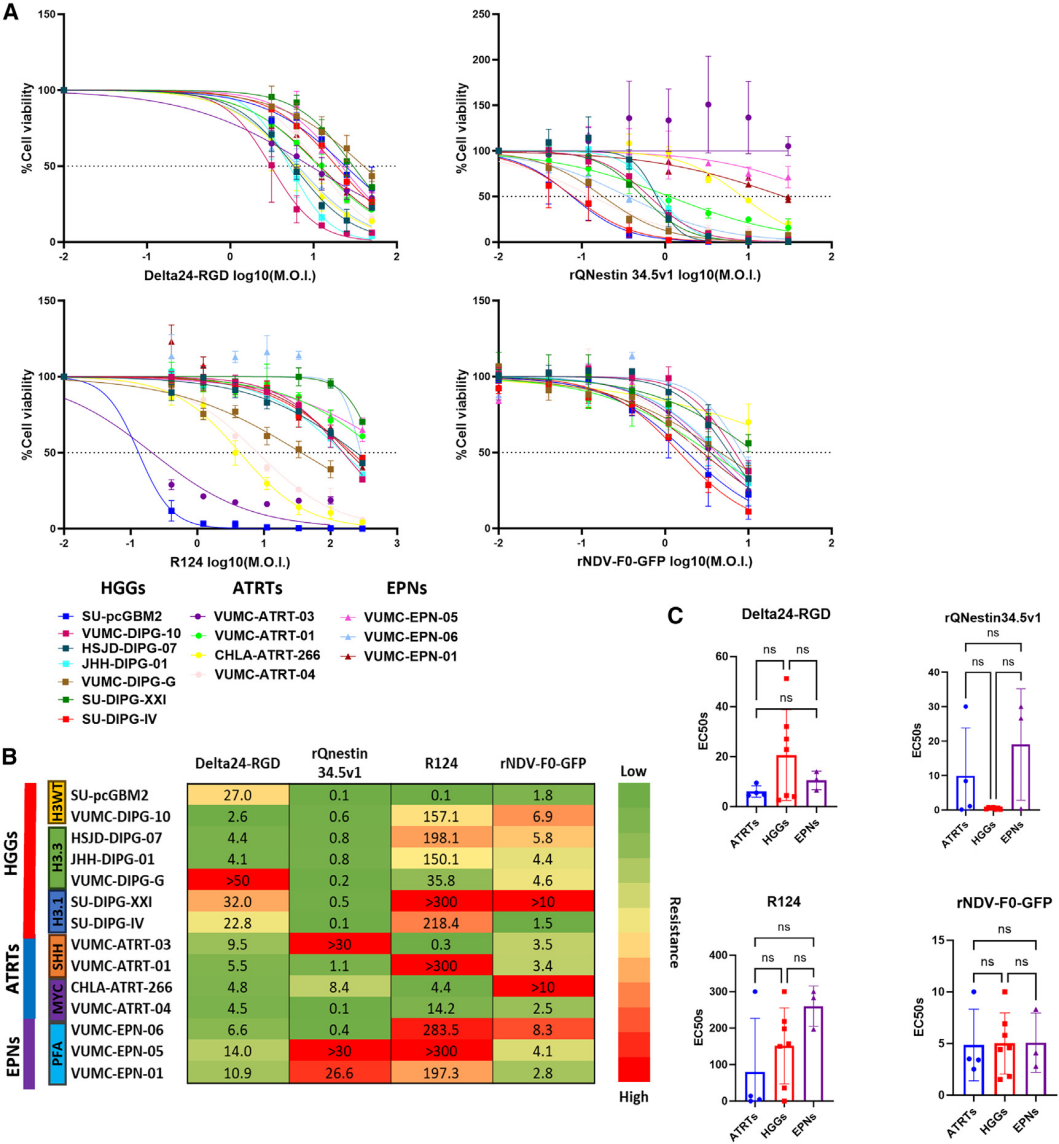
Cell viability assays after OV infection (A) Patient-derived cell cultures were incubated with various dilutions multiplicities of infection (M.O.I.) of Delta24-RGD, rQNestin34.5v1, R124, and rNDV-F0-GFP and cell viability was measured 5 days post OV infection with CTG assay. The data are shown as the percentage of cells alive after infection with Delta24-RGD, rQNestin34.5v1, R124, or rNDV-F0-GFP at the indicated log10 (M.O.I.s) relative to the non-infected cells, with each point corresponding to the mean ± (SD) values of independent CTG assays per cell line (*n* = 2 to 4) (see also Table S1). (B) EC_50_ obtained from (A), the color scaling is independent per OV from green (lowest EC_50_) to red (highest EC_50_). (C) Mean EC_50_ comparisons grouped per tumor entity. Each point in the bar plots corresponds to the EC_50_ of a specific PBT cell culture. For the comparison of the EC50 per tumor entity the non-parametric Kruskal-Wallis test with Dunn’s multiple comparison correction. ns = not statistically significant.

Having demonstrated the expression of genes that serve as entry-related molecules for the four OVs, we screened dose ranges of Delta24-RGD, rQNestin34.5v1, R124, and rNDV-F0-GFP on the PBTs. As depicted in Figure 2A, large variability in response was observed to each OV by each tumor type. To compare the efficacy of the four OVs over the panel of PBTs, we calculated the effective concentration for 50% oncolysis (EC_50_) per OV (Figure 2B; Table S1). As the OVs are each effective within different dose ranges, we only compared the EC_50_ values across PBTs and not across OVs. Mean EC_50_ values for Delta24-RGD ranged from multiplicity of infection (MOI) 2.6 to >50 in HGGs, 4.5 to 9.5 in AT/RTs, and 6.6 to 14 in EPNs (Figure 2B). rQNestin34.5v1 ranged from MOI 0.1 to 0.8 in HGGs, 0.1 to >30 in AT/RTs and 0.4 to >30 in EPNs. R124 ranged from MOI 0.1 to >300 in HGGs, 0.3 to >300 in AT/RTs, and 197.3 to >300 in EPNs. rNDV-F0-GFP ranged from MOI 1.5 to >10 in HGGs, 2.5 to >10 in AT/RTs, and 2.8 to 8.3 in EPNs (Figure 2B). Despite this great variability in OV susceptibility over the PBT panel, every PBT cell culture demonstrated sensitivity to at least one OV, with no significant tumor entity preference noted for any individual OV (Figures 2B and 2C). In summary, these results demonstrate that Delta24-RGD, rQNestin34.5v1, R124, and rNDV-F0-GFP can induce a significant and heterogeneous oncolytic effect in most of the tested PBT cultures. Representative immunofluorescent images of two sensitive and two resistant PBTs on day 5 post infection are depicted as examples of the viral activity of rQNestin34.5v1 and rNDV-F0-GFP in the PBT spheres (Figure S1).

### Correlating OV sensitivity and resistance to basal gene expression

**Figure 3:**
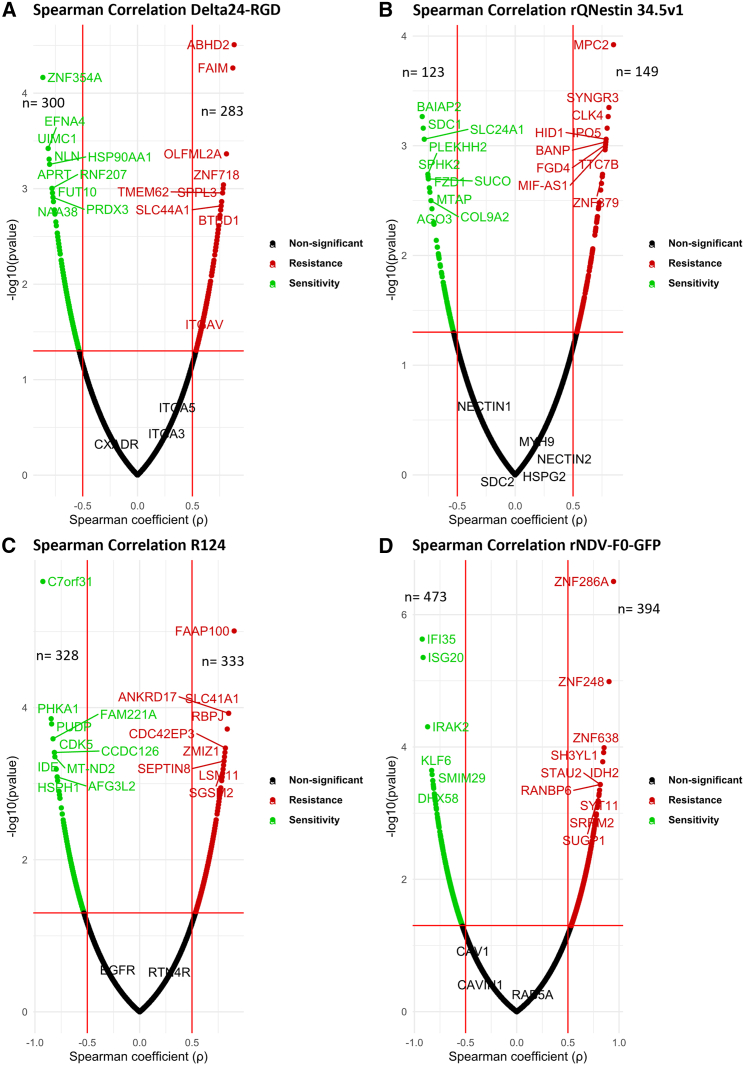
Correlating genes of OV sensitivity and resistance Volcano plots of Spearman’s correlation coefficient (ρ) values demonstrating significantly correlated genes for sensitivity and resistance for (A) Delta24-RGD, (B) rQNestin34.5v1, (C) R124, and (D) rNDV-F0-GFP, resulting from correlating gene expression and EC_50_ values (see also Table S2). The x axis represents the Spearman’s correlation coefficient (ρ), and the y axis represents the −log10 (*p* value). Each dot represents a gene, with black the non-significant (absolute coefficient <0.5 and *p* value <0.05), green the sensitivity-related genes (coefficient < −0.5 and *p* value <0.05), while red the resistance-related genes (coefficient >0.5 and *p* value <0.05). Labeled are the genes related to viral entry using their gene symbol. Volcano plots were created with R (https://www.r-project.org/). Spearman correlation was employed.

To identify genes associated with sensitivity and resistance to the OVs, we performed bulk RNA-sequencing to obtain the transcriptional profiles of these 14 highly heterogeneous PBT sphere cultures in their uninfected state. Using the OVs EC_50_ for the 14 cultures, we assessed Spearman’s correlation coefficient (ρ) and *p* values. A ρ value lower than −0.5 with a *p* value of <0.05 indicated a gene that significantly correlated with sensitivity to the OV, while a ρ value higher than 0.5 with a *p* value <0.05 showed that a gene was associated with resistance (Table S2). Unexpectedly, we found that none of the virus entry-related genes showed a significant correlation with sensitivity to the OVs, except for syndecan 1 (*SDC1*) with ρ < −0.79 (*p* value <0.0007) for rQNestin34.5v1 (Figures 3A–3D).

The number of genes correlated with sensitivity and resistance, respectively, varied greatly among the different OVs (Figures 3A–3D; Table S2). The 10 most correlating genes for Delta24-RGD, rQNestin 34.5v1, R124, and rNDV-F0-GFP are shown in Figures 3A–3D. Interestingly, we observed no overlap between the genes associated with sensitivity and resistance to each OV, suggesting distinct molecular mechanisms underlying these responses.

### Gene Ontology term enrichment of OV correlated genes

**Figure 4:**
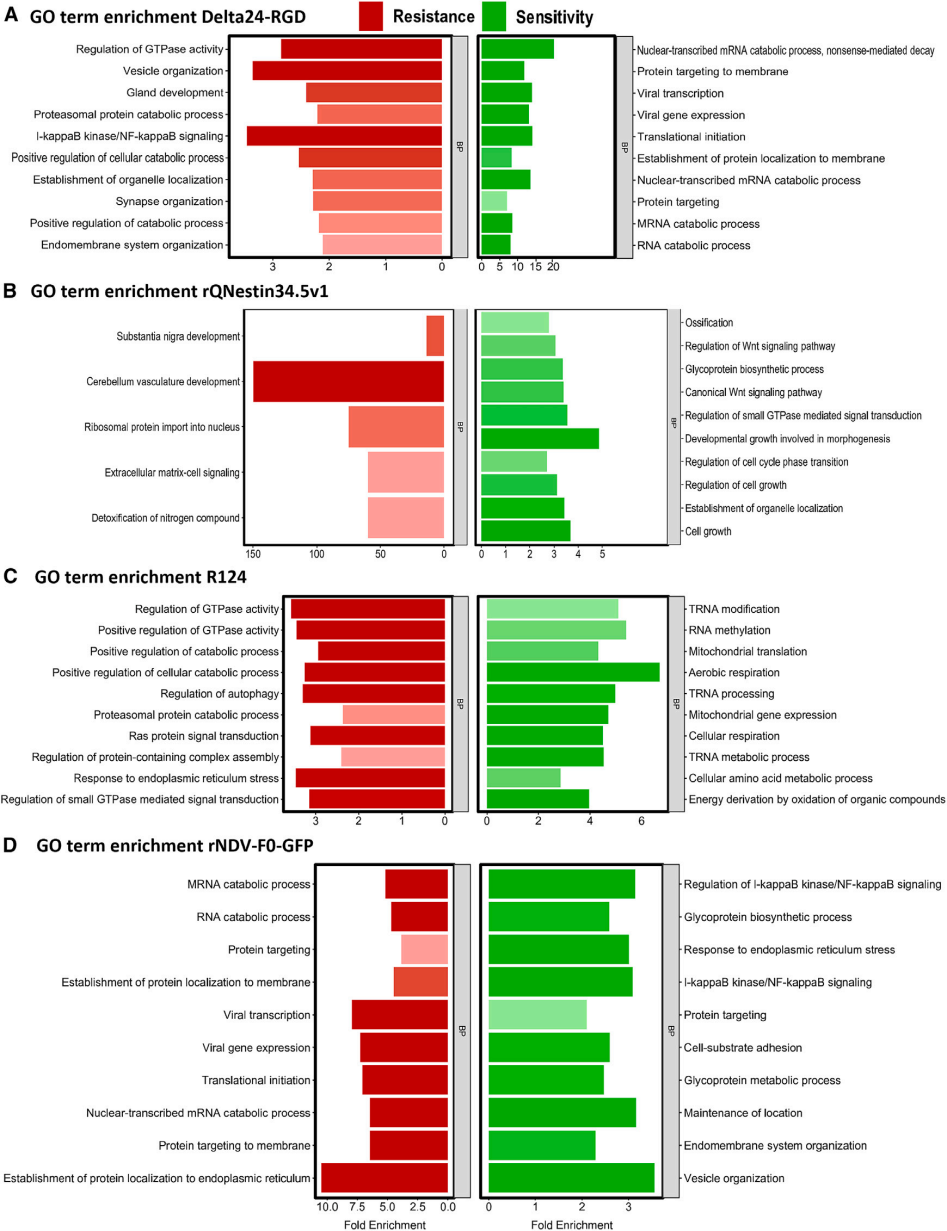
Top 10 most enriched biological processes terms of Gene Ontology The bar plots demonstrate the top 10 Gene Ontology biological processes terms based on the numbers of genes enriched after GO enrichment analysis for Delta24-RGD (A), rQNestin34.5v1 (B), R124 (C), and rNDV-F0-GFP (D). The bar plots are color-coded with green being related to sensitivity and red related to resistance. Significant GO terms (*p* value <0.05) are depicted (see also Table S3). The terms with the lowest *p*-adjusted value are demonstrated with a darker shade of red or green while terms with higher *p* value are depicted with lighter shades of red or green, respectively. Bar plots were created with R (https://www.r-project.org/). The *p*-adjusted value was calculated through the FDR method.

To characterize the biological processes associated with sensitivity and resistance to the different oncolytic viruses, we analyzed the sets of genes that showed significant correlations with sensitivity and resistance (Table S2). We performed Gene Ontology (GO) term enrichment analysis to identify distinct biological processes linked to susceptibility to each specific OV (Figures 4A–4D; Table S3). For Delta24-RGD sensitivity, the related genes were enriched in terms associated with viral transcription and viral gene expression (Figure 4A). Resistance-related genes were enriched in terms related to NF-κB signaling and vesicle organization (Figure 4A). The sensitivity to rQNestin34.5v1 was associated with cellular growth, WNT signaling, and glycoprotein synthesis, whereas resistance was related to cerebellum vasculature development and ribosomal protein import into the nucleus (Figure 4B). Genes associated with R124 sensitivity were enriched in terms related to mitochondrial activity and RNA processing, R124 resistance was linked to terms such as positive GTPase regulation, autophagy, and Ras protein signaling (Figure 4C). rNDV-F0-GFP sensitivity-related genes were enriched in terms related to NF-κB signaling, glycoproteins, and adhesion (Figure 4D). Conversely, resistance to rNDV-F0-GFP was linked to terms related to transcription and translation (Figure 4D).

### Validation of the molecular profiles of sensitivity and resistance

Correlation of EC_50_ values with RNA expression of the PBT cultures before infection provided us with a list of genes that enrich for biological functions relating to each OV. To assess the potential of the correlated genes for sensitivity and resistance to predict OV cytotoxic potential, we extended our screening with four additional PBT sphere cultures (KNS-42, OPBG-GBM-001, VUMC-DIPG-11, VUMC-DIPG-F), referred to as validation cultures. Their molecular signatures were compared with previously utilized PBT cultures, referred to as confirmation cultures, by unsupervised clustering using the gene expression profiles for sensitivity and resistance (Table S2) and by *in vitro* OV sensitivity testing.

**Figure 5:**
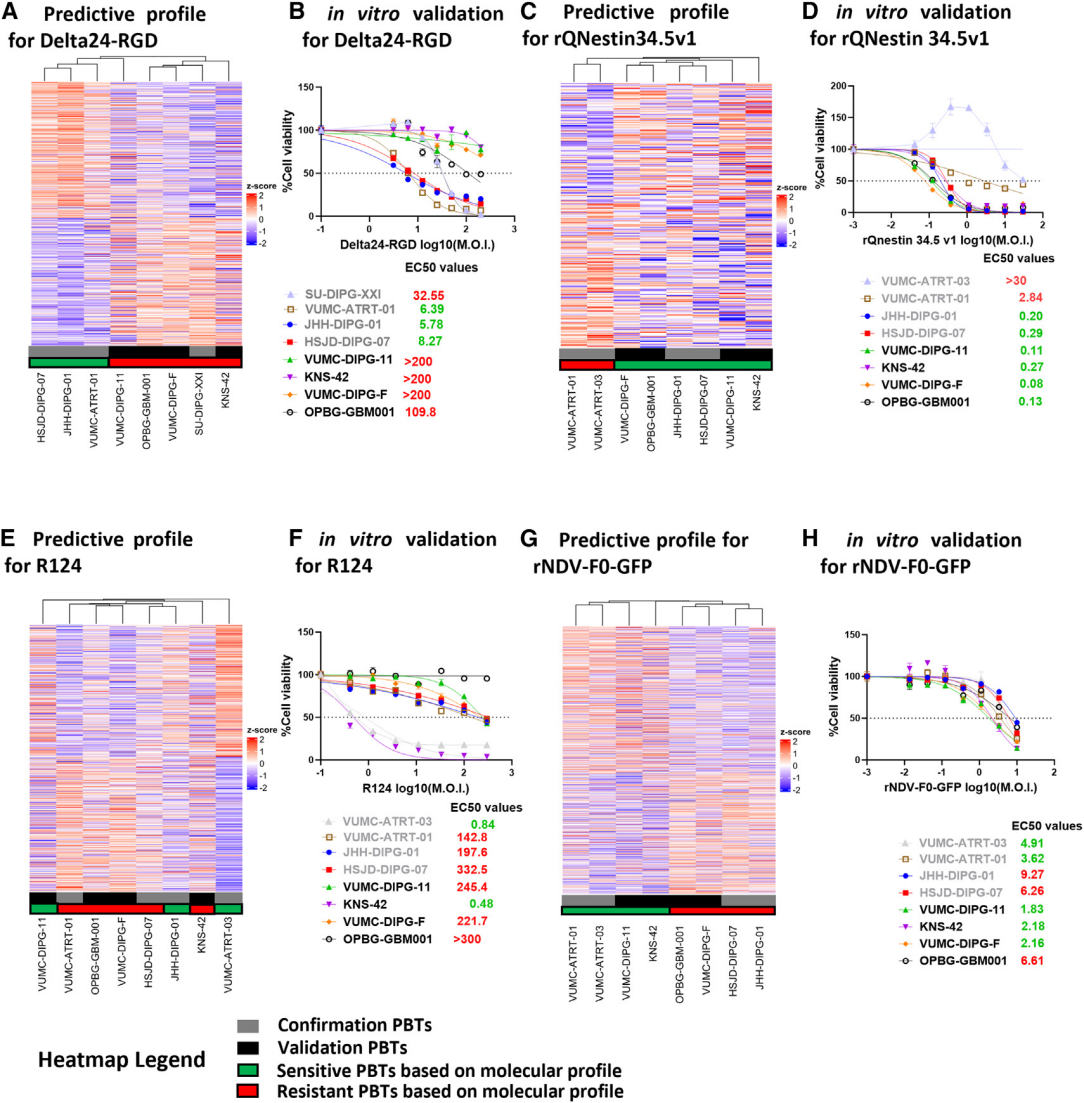
*In vitro* validation of OV sensitivity signatures Each heatmap represents the expression of genes that significantly correlated with Delta24-RGD (A), rQNestin34.5v1 (C), R124 (E), and rNDV-F0-GFP (G) resistance and sensitivity. Each column represents a PBT culture cluster based on the average expression of the genes. Each line represents the *Z* score expression levels of all correlated genes for resistance and sensitivity. Cell viability graphs of four confirmation PBT cultures in gray and four newly tested validation PBT cultures in black after 5-day incubation with Delta24-RGD (B), rQNestin34.5v1 (D), R124 (F), and rNDV-F0-GFP (H). Cell viability was measured for the validation of the predictive profiles as described in Figure 2A. Each point represents the mean ± (SD) (*n* = 3). Heatmaps were plotted in R (https://www.r-project.org/).

Based on the signature for Delta24-RGD sensitivity, the molecular profiles of the four PBT validation cultures clearly clustered with the resistant confirmation culture SU-DIPG-XXI, while clustering the sensitive confirmation cultures JHH-DIPG-01, HSJD-DIPG-07, and VUMC-ATRT-01 together, thus predicting the resistance of all validation cultures (Figure 5A). Indeed, upon *in vitro* testing for Delta24-RGD, the four validation cultures proved to be highly resistant, with EC_50_ values higher than 50 (Figure 5B). For rQNestin34.5v1, the molecular signatures of all four validation cultures clustered together with those of the sensitive confirmation cultures JHH-DIPG-01 and HSJD-DIPG-07, while the resistant confirmation cultures VUMC-ATRT-03 and VUMC-ATRT-01 clustered separately (Figure 5C). Again, dose-response testing with rQNestin34.5v1 resulted in EC_50_ values lower than 0.3 in all four validation cultures, in accordance with predicted sensitivity (Figure 5D). In the case of R124, the unsupervised clustering of the molecular signatures did not fully separate the sensitive from the resistant cultures (Figure 5E). Of the three predicted resistant validation cultures OPBG-GBM-001, VUMC-DIPG-F, and KNS-42, the latter revealed a highly sensitive *in vitro* response to R124 with an EC_50_ of 0.48. Inversely, the validation cultures VUMC-DIPG-11 that demonstrated a sensitive profile revealed an EC_50_ value of 221.7 (Figure 5F). The remainder of the validation cultures (VUMC-DIPG-F and OPBG-GBM-001) clustered with the resistant VUMC-ATRT-01 and HSJD-DIPG-07 (Figure 5E) and *in vitro* validation indeed demonstrated resistance to R124 (Figure 5F). Finally, the molecular profiles for rNDV-F0-GFP also resulted in two clear clusters, delineating predicted sensitive and resistant PBT cultures to rNDV-F0-GFP (Figure 5G). *In vitro* validation showed that the expression profiles successfully predicted the relative sensitivity of the validation cultures KNS-42 (EC_50_ = 2.18), VUMC-DIPG-11 (EC_50_ = 1.8), as well as the resistance of OPBG-GBM-001 (EC_50_ = 6.61), while failing to predict the sensitivity of VUMC-DIPG-F with EC_50_ = 2.16 (Figure 5H). Taken together, the analysis shows that the identified signatures of OV sensitivity can act as biomarkers for predicting the relative sensitivity or resistance of PBT cell cultures to OV-induced oncolysis, in particular for Delta24-RGD and rQnestin34.5v1.

## Discussion

Several OVs have been investigated both in preclinical and clinical studies for treatment of PBTs, showing a beneficial safety profile and promising efficacy, either as monotherapy or when combined with radiotherapy or chemotherapy.^51^^,^^52^ Ongoing clinical investigations using OVs in PBTs remain limited to medulloblastoma and HGG (AdVs: NCT03178032, NCT05717712, NCT05717699, NCT0458533. HSVs: NCT02457845, NCT03911388, NCT04482933, NCT02031965. RVs:NCT02444546. NDVs: NCT01174537. Measles viruses: NCT02962167. PVs: NCT03043391), while in preclinical investigations of DNX-2401 AT/RTs were included.^19^^,^^42^^,^^51^^,^^53^^,^^54^^,^^55^ No prior study has included EPNs with OVs. Here, we assessed the oncolytic efficacy of Delta24-RGD, rQNestin34.5v1, R124, and rNDV-F0-GFP for HGGs, AT/RTs, and EPNs.

Our investigation demonstrated the expression of viral entry genes for the different OVs by each tumor entity and the effective oncolysis of PBTs. The use of Delta24-RGD on HGGs and AT/RTs resulted in EC_50_ ranges similar to those previously reported by Alonso et al.,^42^^,^^53^ demonstrating the reproducibility of our screening process. Interestingly, Delta24-RGD showed high oncolytic potency against many AT/RTs and EPNs with MOIs below 10. HGGs also demonstrated a general sensitivity to rQNestin34.5v1 with MOIs below 1. However, when comparing the groups, none of the OVs displayed significant oncolytic potency to any individual PBT entity.

The use of patient-derived tumor sphere cultures has become the gold standard in drug screening and has contributed to the identification of cell-intrinsic factors that affect therapeutic efficacy, while better reflecting clinical responses.^38^^,^^41^^,^^56^^,^^57^ As we observed a considerable variability in EC_50_ ranges across PBTs, even within the same tumor entity, we decided to capitalize on tumor cell-intrinsic factors that dictate sensitivity or resistance to each OV by correlating the transcriptome of the PBT cultures with their EC_50_ values. As each OV employs different mechanisms in the host cells to induce its cytotoxic action, this approach could potentially yield biomarkers of response. Indeed, a recent systematic analysis of all publications relating to oncolytic virus resistance demonstrated that the main source of resistance to OV therapy originates from different cell-intrinsic factors, including cell survival, heterogeneity, hypoxia, growth factors, epigenetic factors, viral entry, and interferon responses.^58^

Our analysis provided a list of genes correlating either with sensitivity or resistance to each OV. Interestingly, of the 18 viral entry-related genes, only the expression of the HSV entry receptor *SDC1*, which was previously shown to greatly influence the spread of HSV-1,^59^ significantly correlated with sensitivity. The relevance of the correlated genes in our study became more apparent when they were mapped to GO biological processes relevant to each OV. The GO terms for Delta24-RGD align with the well-known importance of the interplay between transcription, translation, and AdV-host interactions across different timepoints of AdV5 infection, early points of infection have demonstrated to be heavily dependent on chromatin acetylation.^60^^,^^61^^,^^62^^,^^63^ Splicing-related terms were also correlated with sensitivity, a process first discovered in AdVs and essential for infection cycle.^64^ In the case of resistance, terms related to NF-κB regulation were also consistent with previous studies indicating that inhibition of NF-κB enhances the oncolytic cytotoxicity of AdVs.^65^ Terms related to hypoxia together with increased expression of *HIF-1A* correlated with Delta24-RGD resistance, a process known to negatively affect the cytolytic effect and production of AdV5 without affecting viral entry in all cells tested.^66^ In addition to these terms, the gene *Mx1(MxA)* correlated with Dela24-RGD resistance, as previously reported in adenovirus resistance.^67^

Glycoproteins are essential for HSV entry and GO terms as “glycoprotein biosynthetic process,” “protein glycosylation,” and “aminoglycan biosynthesis” were identified, thus highlighting their importance as a cell-intrinsic factor affecting PBT sensitivity to rQNestin34.5v1.^43^^,^^68^^,^^69^ Furthermore, the term “exosomal secretion” was also related to sensitivity. Exosomes have been demonstrated as an important tool for HSV-1 release from infected cells and spreading to uninfected cells.^70^ WNT/β-catenin signaling has been associated with HSV-1 productive infection and our analysis also associated to this pathway with PBT sensitivity to rQNestin34.5v1.^71^

The dependence of R124 to both GTPase activity and Ras pathway signaling was clearly demonstrated in GO terms like “negative regulation of Ras protein signaling” and “positive regulation of GTPase activity,” which correlated with PBT resistance to R124.^19^^,^^26^^,^^27^^,^^28^ Briefly, GTPase activity has been reported to downregulate the Ras protein superfamily, as it hydrolyses the active RAS-GTP to its inactive form, while Ras over-activation is crucial for promoting RV activity.^26^^,^^27^^,^^28^^,^^72^^,^^73^ In addition, the analysis revealed the relevance of mitochondrial activity in the context of RV, which aligns with its reported importance in inducing apoptosis upon RV infection.^74^

In the case of rNDV-F0-GFP, sensitivity GO terms such as “vesicle organization,” “vesicle coating,” “regulation of endocytosis,” and “endosome to lysosome transport” highlight the importance of the different entry mechanisms as a factor of successful oncolysis. The ability of NDV to utilize multiple endocytosis pathways for cell entry have previously been demonstrated.^49^^,^^75^^,^^76^ Recent observations have indicated that NF-κB-triggered JNK activation promotes apoptosis and inflammation, supporting rNDV-F0-GFP proliferation and validating the GO terms “IRE1-mediated unfolded protein response” and “I-kappaB kinase/NF-κB signaling.”^77^^,^^78^^,^^79^ In addition to those terms, the gene *ATG5* significantly correlated with PBT sensitivity to rNDV-F0-GFP, aligning with previously reported siRNA silencing of *ATG5* affecting NDV production in U251 glioma cells.^80^

Overall, our analysis uncovered a number of genes and GO terms of importance in different stages of the infection cycles of the OV species applied. These findings provide insights into the underlying mechanisms that affect oncolytic efficacy of OVs in pediatric brain tumors (PBTs).

Importantly, the predictive value of the correlated genes was validated in an independent set of PBT cultures with the expression profiles for Delta24-RGD and rQNestin34.5v1, correctly clustering and predicting the relative sensitivity or resistance of all four new cultures. In addition, the markers for rNDV-F0-GFP were able to correctly predict sensitivity of three of the four newly tested PBT cultures. For R124, the signature genes were not able to predict as effectively the relative resistance/sensitivity of the newly tested cultures.

Validation of the correlated genes as biomarkers for sensitivity or resistance to specific OVs *in vitro*, represents a first step toward a tailored OV therapy. However, it is essential to evaluate the predictive value of the identified molecular profiles on a larger, independent cohort of pediatric brain tumor samples. Moreover, our *in vitro* model system does not support assessment of the other key mode of action of OVs, namely generation of an anti-tumor immune response. More advanced *ex vivo* models, incorporating the tumor microenvironment and/or immune components, such as autologous tumor/PBMC co-cultures, may aid in acquiring further insight into the relationship between OV-induced oncolysis and degree of immune stimulation.^81^ Furthermore, concurrently screening of patient-derived tumor samples alongside clinical investigations may assist in the further validation and refinement of the identified molecular signatures.

In conclusion, our study demonstrates that Delta24-RGD, rQNestin34.5v1, R124, and rNDV-F0-GFP hold oncolytic potential for multiple entities of PBTs. By screening multiple patient-derived cell cultures, we were able to identify cell-intrinsic factors that relate to sensitivity and resistance to the four selected OVs. In addition, the predictive power to oncolytic efficacy *in vitro* of those genes was validated. As we continue to explore and validate such biomarkers in future translational research, we move closer to developing personalized medicine and improving the efficacy of oncolytic virus-based therapies for PBT patients.

## Materials and methods

### Patient-derived cell cultures

HSJD-DIPG-07 (H3.3K27M, DMG) was a kind gift from Dr. Carcaboso (Hospital Sant Joan de Déu, Barcelona, Spain)^82^; JHH-DIPG-01 (H3.3K27M, DMG) were provided by Dr. Raabe (John Hopkins Hospital, Baltimore, MD, USA)^83^; SU-DIPG-IV (H3.1K27M, DMG), SU-DIPG-XXI (H3.1K27M, DMG), and SU-pcGBM2 (H3WT, GBM) were provided by Dr. Monje (Stanford University, Stanford, CA, USA)^84^; CHLA-ATRT-266 (MYC, ATRT) was obtained from the American Type Culture Collection (ATCC)^85^; KNS-42 (H3.3G34, DMG) was obtained from the JCRB (Japan Cancer Research Resources) cell bank^86^; OPBG-GBM-001 (H3WT, GBM) was provided by Dr. Vinci (Bambino Gesú Children’s Hospital); while VUMC-DIPG-10 (H3WT, DMG), VUMC-DIPG-G (H3.3K27M, DMG), VUMC-DIPG-11 (H3.3K27M, DMG), VUMC-DIPG-F (H3.3K27M, DMG), VUMC-ATRT-03 (SHH, ATRT), VUMC-ATRT-01 (SHH, ATRT), VUMC-ATRT-04 (MYC, ATRT), VUMC-EPN-06 (PFA, EPN), VUMC-EPN-05 (PFA, EPN), and VUMC-EPN-01 (PFA, EPN) were established from autopsy or resection material at the Amsterdam UMC (Vrije University Medical Center of Amsterdam, Amsterdam, the Netherlands).^38^^,^^56^^,^^87^^,^^88^ Previously characterized and known mutations of the samples are shown in detail according to Table S4 also found in (shinyapps.io).^41^^,^^89^ All patient material was collected according to national and institutional guidelines and in accordance with the declaration of Helsinki.

HSJD-DIPG-07, JHH-DIPG-01, SU-DIPG-IV, SU-DIPG-XXI, SU-pcGBM2, CHLA-ATRT-266, VUMC-DIPG-10, VUMC-DIPG-G, VUMC-ATRT-03, VUMC-ATRT-01, KNS-42, OPBG-GBM-001, VUMC-DIPG-11, and VUMC-DIPG-F were cultured at 37^o^C and 5% CO_2_ in Tumor Stem Medium (TSM) consisting of 48% Neurobasal-A medium (Thermo Fisher, #10888022, Amsterdam, the Netherlands), 48% Dulbecco’s modified Eagle’s medium (DMEM)/F12 with Phenol Red without glutamine (Thermo Fisher, #31330095, Amsterdam, the Netherlands), 1% HEPES 1M (Thermo Fisher, #15630-080, Amsterdam, the Netherlands), 1% MEM Non-essential amino acid solution (Thermo Fisher, #11140050, Amsterdam, the Netherlands), 1% Sodium pyruvate 100 mM (Thermo Fisher, #11360039, Amsterdam, the Netherlands), 1% Glutamax (Thermo Fisher, #35050038, Amsterdam, the Netherlands) (TSM base). TSM base was supplemented with 2% B27 without vitamin A (Thermo Fisher, #12587010, Amsterdam, the Netherlands), 20 ng/mL human Epidermal Growth Factor (Peprotech, #AF-100-18B-1MG, Amsterdam, the Netherlands), 20 ng/mL human Basic Fibroblast Growth Factor (Peprotech, #AF-100-18B-1MG, Amsterdam, the Netherlands), 10 ng/mL human Platelet-derived Growth Factor AA (Peprotech, #100-13A-250μG, Amsterdam, the Netherlands), 10 ng/mL human Platelet-derived Growth Factor BB (Peprotech, #100-14B-250μG, Amsterdam, the Netherlands), 5,000 U/mL Heparin and 1% penicillin/streptomycin (Sigma Aldrich, P0781-100ML, Amsterdam, the Netherlands) (Complete TSM).

VUMC-ATRT-04, VUMC-EPN-06, VUMC-EPN-05, and VUMC-EPN-01 were cultured at 37^o^C and 5% CO_2_ in TSM base supplemented with 10% heat-inactivated FBS (Sigma Aldrich, F0804, Amsterdam, the Netherlands) and 1% penicillin/streptomycin.

Cells were only used when confirmed mycoplasma negative with MycoAlert Mycoplasma Detection kit (Lonza, LT07-318) and short-tandem repeat analysis with GenePrint 10 system (Promega, B9510, Leiden, the Netherlands) was performed to ensure cell line identities.

### Viruses

Delta24-RGD, rQNestin34.5v1, R124, and rNDV-F0-GFP were produced as previously described.^25^^,^^30^^,^^34^^,^^90^ Delta24-RGD viral stocks were titrated on HEK 293 cells using the Adeno-X Rapid Titer Kit (Takara,#632250), 12-well plates of HEK 293 cells were incubated in DMEM and 10% FBS, while 10-fold serial dilutions of the AdV were prepared and incubated with the cells for 48 h, the medium was then removed and cells were left to dry in hood for 5 min. The HEK 293 cells were then fixed with 100% methanol and incubated at −20^o^C for 10 min and rinsed three times. Mouse anti-hexon antibody (Takara,#632250) 1:1,000 in phosphate-buffered saline (PBS) was incubated with the cells for 1 h at 37^o^C and then rinsed and incubated with Rat anti-mouse (HRP-conjugated) (Takara,#632250) 1:500 for 1 h at 37^o^C and rinsed. DAP working solution was added to each well for 10 min at RT and washed. The infectious units (iu/mL) were calculated with optically counting the brown/black positive cells with the following formula: (infected cell/field) × (field/well))⁄(volume virus (mL) × (dilution factor). The physical titer of Delta24-RGD was obtained through OD_260_ by lysing the viral stock in a 20-fold dilution in lysis solution (0.1% SDS, 10 mM Tris-Cl [pH 7.4], 1 mM EDTA), incubating at 56^o^C for 10 min, after which the disrupted virus solution was placed in a cuvette and the OD_260_ was determined. The concentration of the AdV was calculated in vp/mL by OD260 reading × dilution factor × 1.1 × 10^12^ = vp/mL.

rQNestin34.5v1 was titrated by plaque assay on Vero cells. Briefly, six-well plates of Vero cells were incubated for a day at 37^o^C at 5% CO_2_ in DMEM and 10% FBS. The following day, 10-fold dilutions of the HSV stock were prepared (10^−2^ to 10^−10^) and left for 60 to 90 min in the incubator with frequent rocking of the plates. After 90 min, the virus inoculum was removed and the wells were overlaid with 1% methylcellulose and incubated for 3–5 days until defined plaques were formed. The methylcellulose was then removed and the wells were stained with 1% crystal violet for 5 min at RT prior to rinsing and air drying. The number of plaque-forming units (PFU)/mL was determined by (the average number of plaques of each dilution multiplied by a factor of 10). The physical titer of the virus was measured as genome copies (G.C./mL) by quantifying the average G.C. of UL5 (F: 5′-GTG ATG CGA CTG GCG TTG G-3′, R: 5′-CAG TTT GTG GAC CGC TTT GT-3′) and glycoprotein D (US6) (F: 5′-GCG TGT TTA CCA CAT TCA GCC-3′, R: 5′-TCC GTC CAG TCG TTT ATC TTC A-3′) genes using standard curves for UL5 or US6 originating from 10-fold serial dilutions of plasmid containing UL5/US6 target sequences quantified by ABI-Plus 7000 Sequence Detector (ABI, USA) and analyzed using Sequence Detector V1.6 (PE Applied Biosystems, USA).

For R124, RV stocks were serially diluted in DMEM and 2% FBS and infected near-confluent HER 911 cells in six-well plates for 2-h incubation at 37^o^C, the medium was replaced with minimal essential medium (MEM), 12.5 mM MgCl_2_, 2 mM GlutaMAX, and 0.5% agarose and plaques were counted six days post infection and PFU/mL was determined by (average number of dilutions × 10). The physical titer of R124 was measured through OD_260_ by lysing the viral stock as was described for Delta24-RGD. Vp/mL was estimated by empirical determined relation: 5.42 OD_260_ = 1mg of RV = 1.13 × 10^13^ RV particles.^91^

For rNDV-F0-GFP, viral stocks were titrated by endpoint dilution assay in Vero cells previously incubated overnight in DMEM and 2% FBS in 48-well plates. Ten-fold serial dilutions of the virus were prepared and used to infect the Vero cells for 2 h at 37^o^C, followed by addition of medium and monitoring cytopathic effects (CPE) for 1 to 4 weeks. Titers were calculated as tissue culture infectious dose (TCID) required to infect 50% of the cell monolayers by visualizing CPE and using the method of Reed and Muench.^92^

### Cell viability assay

Single-cell suspensions were collected after cells were treated with accutase (Merck, A6964-100ML, Amsterdam, the Netherlands) and washed with PBS. Then, 2,000 cells/well were seeded in triplicate onto 96-well flat-bottom plates in 100 μL of the respective cell line’s fresh culture media and left to incubate at 37^o^C and 5% CO_2_ for 24 h. The cells were then infected with increasing doses of virus and left to incubate at 37^o^C and 5% CO_2_ for 5 days. The concentration of the OVs is defined as MOI, which constitutes the number of infectious viral particles per cell. MOI ranges were for Delta24-RGD (MOI 50, 25, 12.5, 6.25, 3.125), for rQNestin34.5v1 (MOI 30, 10, 3.3, 1.1, 0.37, 0.12, 0.04), for R124 (MOI 300, 100, 33, 11, 3.7, 1.23, 0.41), and for rNDV-F0-GFP (MOI 10, 3.3, 1.1, 0.37, 0.12, 0.04, 0.01). During infections with rQNestin35.4v1, culture medium without heparin was applied in the assay to prevent non-cellular binding of HSV particles to soluble heparin, while in the cases in which FCS-supplemented culture medium was applied, the FCS concentration was reduced to 2% on the day of infection to prevent FCS interference with viral attachment. On day 5, infection was stopped, and cells were incubated for 10 min with CellTiter-Glo 2.0 assay reagent (Promega, #G924A, Leiden, the Netherlands), then the mixes were transferred into new clear flat-bottom black 96-well plates (Greiner, #655076, Mannheim, Germany) and luminescence was measured using a Tecan infinite M200 plate reader (Tecan) using the icontrol 1.10 software (Tecan). Background luminescence in the corresponding medium without cells was subtracted from experimental values. Values were normalized for calculating percentage of viable cells taking the values of uninfected cells as 100%. The mean values of the normalized data from two to four independent experiments per virus per cell line were used in a dose-normalized response with a variable slope and from the non-linear fit the effective concentrations needed to kill at least 50% of the cells (EC_50_) for each virus was calculated using GraphPad Prism 9.

### Immunofluorescence imaging

Cells infected with rQNestin34.5v1 and rNDV-F0-GFP in a 96-well plate were imaged after 5 days of infection with MOI 3.3 and 1.1, respectively, using EVOS M5000 Imaging System (Invitrogen, USA) at ×100 magnification.

### RNA-sequencing and analysis

Total RNA of non-infected cell lines was extracted and collected before the viability assay, using the miRvana miRNA isolation kit (Ambion, #AM1560, Landsmeer, the Netherlands) according to the manufacturers protocol. RNA quality and purity were assessed by the Agilent 2100 Bioanalyzer system using the Agilent RNA6000 Nano kit (Agilent, #5067-1511, CA, USA) and samples with RIN value of <7 were excluded from sequencing. The sample preparation was performed according to the protocol "NEBNext Ultra Directional RNA Library Prep Kit for Illumina" (NEB #E7420 S/L), briefly using oligo-dT magnetic beads mRNA was isolated from total RNA, followed by fragmentation and cDNA synthesis. After ligation of the sequencing adapters and PCR amplification, the quality and yield were measured with Fragment Analyzer. Clustering and DNA sequencing were performed with NovaSeq6000 following the manufacturer’s protocol. Sequence reads were trimmed using cutadapt v2.10 removing adapter sequences. For each sample, the trimmed reads were mapped to the human GRCh38.p13 reference genome based on Burrow-Wheeler Transform using the default settings of STARv2.5.4. The BAM files were sorted and indexed with the samtools v1.10 package. All sequencing, adapter removal, trimming, and mapping and feature counting were performed by GenomeScan (Leiden, the Netherlands). The count matrix was loading in EdgeR package the gene counts with the same gene symbol were summarized using the R function “aggregate.”^93^ Then using the R function “filterByExpr,” genes with less than 10 counts and not expressed in 70% of the samples or a total count of less than 15 counts across all samples were filtered out. The filtered genes were normalized through a trimmed mean of M-values (TMM) and counts per million (CPM) were computed followed by a log2 (CPM) transformation to be used in downstream analysis.

### Viral sensitivity correlation

The correlation of the transcription level of genes and EC_50_ values was calculated by Spearman correlation per OV based on normalized gene expression of the tested cell lines. The correlation was executed using the “cor.test” function from the basic R package for a univariate Spearman correlation and genes with a *p* value <0.05 and ρ coefficient >0.5 were defined as significant genes correlating with resistance whereas genes with ρ coefficient <−0.5 were defined as genes correlating with sensitivity.

### GO enrichment analysis

GO enrichment analysis was performed separately for the resistance correlated genes and sensitivity-related genes using the R package “cluster profiler.”^94^ The significant genes correlated with the sensitivity and resistance identified from the correlation analysis were used to query the GO database,^95^ respectively. GO terms with a *p-*adjusted value (FDR method) < 0.05 were considered significantly enriched by the correlating genes in the corresponding groups.

### Statistical analysis

For the *in vitro* experiments, the data were visualized as the mean ± standard deviation (SD), and the comparisons of mean EC_50_ values between the tumor entity groups were performed using a non-parametric Kruskal-Wallis test with Dunn’s multiple comparison correction. All statistics for EC_50_ calculation and comparisons were performed using GraphPad Prism 9, while *p* values, ρ coefficients for the gene expression, correlation, and GO enrichment were performed using R.

## Data and code availability

The expression data supporting the findings of this study are available in the GEO database with ascension number GEO: GSE260516.

## Supplemental information

Document S1. Figure S1 and Tables S1 and S4–S6

Table S2. Significantly correlating genes

Table S3. Gene Ontology enrichment

Document S2. Article plus supplemental information
