# Supplementary material for: The heterogeneous sensitivity of pediatric brain tumors to different oncolytic viruses is predicted by unique gene expression profiles
Source: Mol Ther Oncol. 2024 Apr 15;32(2):200804. doi: 10.1016/j.omton.2024.200804 (PMC11060958; doi:10.1016/j.omton.2024.200804)
Supplement: Document S1. Figure S1 and Tables S1 and S4–S6 [file mmc1.pdf]

OMTON, Volume *32*

Supplemental information

The heterogeneous sensitivity of pediatric brain tumors to different oncolytic viruses is predicted by unique gene expression profiles

Konstantinos Vazaios, Eftychia Stavrakaki, Lisette B. Vogelezang, Jie Ju, Piotr Waranecki, Dennis S. Metselaar, Michaël H. Meel, Vera Kemp, Bernadette G. van den Hoogen, Rob C. Hoeben, E. Antonio Chiocca, William F. Goins, Andrew Stubbs, Yunlei Li, Marta M. Alonso, Friso G. Calkoen, Esther Hulleman, Jasper van der Lugt, and Martine L.M. Lamfers

Table S1. EC50 values

	Delta24-RGD EC50s[a]		
Cell-culture	Best-fit values EC50	95% CI (profile likelihood) EC50	R squared
HSJD-DIPG-07	4.426	2.450 to 6.473	0.8976
VUMC-DIPG-10	2.638	2.096 to 3.091	0.9655
VUMC-DIPG-G	51.15	34.92 to 82.13	0.86
SU-DIPG-XXI	31.97	25.07 to 40.99	0.9449
SU-DIPG-IV	22.78	19.31 to 27.16	0.9509
SU-pcGBM2	26.99	18.38 to 42.54	0.8031
JHH-DIPG-01	4.083	3.704 to 4.486	0.9898
VUMC-ATRT-03	9.47	5.923 to 14.02	0.9254
VUMC-ATRT-01	5.482	2.286 to 10.06	0.7782
CHLA-ATRT-266	4.841	3.428 to 6.457	0.9373
VUMC-ATRT-04	4.47	1.604 to 8.915	0.7757
VUMC-EPN-06	6.61	5.768 to 7.517	0.9805
VUMC-EPN-05	14.03	8.664 to 22.92	0.8827
VUMC-EPN-01	10.85	5.693 to 19.64	0.8732

a. Effective concentration needed to kill 50% of the cells

	rQNestin 34.5v1 EC50s[a]		
Cell-culture	Best-fit values EC50	95% CI (profile likelihood) EC50	R squared
HSJD-DIPG-07	0.7853	0.5549 to 1.044	0.9357
VUMC-DIPG-10	0.6459	0.5720 to 0.7291	0.9926
VUMC-DIPG-G	0.1786	0.1412 to 0.2262	0.9716
SU-DIPG-XXI	0.5347	0.4937 to 0.5793	0.9968
SU-DIPG-IV	0.07856	0.04815 to 0.1269	0.8893
SU-pcGBM2	0.07621	0.04999 to 0.1149	0.912
JHH-DIPG-01	0.8354	0.7122 to 0.9788	0.9851
VUMC-ATRT-03	out of bounds	???	???
VUMC-ATRT-01	1,121	0.7109 to 1.783	0.9276
CHLA-ATRT-266	8,412	6.128 to 11.72	0.9376
VUMC-ATRT-04	0.1326	0.08301 to 0.2102	0.9163
VUMC-EPN-06	0.3537	0.2498 to 0.5005	0.9541
VUMC-EPN-05	89.96	22.75 to 148240976	0.3554
VUMC-EPN-01	26.62	17.64 to 46.68	0.9278

a. Effective concentration needed to kill 50% of the cells

R124 EC50s[a]

Cell-culture	Best-fit values EC50	95% CI (profile likelihood) EC50	R squared
HSJD-DIPG-07	198.1	133.4 to 331.1	0.8738
VUMC-DIPG-10	157.1	121.4 to 212.2	0.9291
VUMC-DIPG-G	35.77	21.64 to 68.00	0.8925
SU-DIPG-XXI	471.6	??? to 2977	0.5478
SU-DIPG-IV	218.4	158.9 to 317.5	0.9495
SU-pcGBM2	~ 0.1357	(Very wide)	0.9748
JHH-DIPG-01	150.1	111.7 to 212.4	0.9237
VUMC-ATRT-03	0.3023	0.1123 to 0.7260	0.644
VUMC-ATRT-01	717.9	354.1 to 2414	0.8088
CHLA-ATRT-266	4,408	3.342 to 5.834	0.9693
VUMC-ATRT-04	8.26	5.027 to 13.92	0.9142
VUMC-EPN-06	283.5	189.6 to 575.1	0.5468
VUMC-EPN-05	461.1	309.2 to 865.3	0.9063
VUMC-EPN-01	197.3	111.5 to 482.2	0.7908

a. Effective concentration needed to kill 50% of the cells

rNDV-F0-GFP EC50s[a]

Cell-culture	Best-fit values EC50	95% CI (profile likelihood) EC50	R squared
HSJD-DIPG-07	5.819	4.473 to 7.788	0.9137
VUMC-DIPG-10	6.88	5.573 to 8.697	0.9314
VUMC-DIPG-G	4.596	3.102 to 7.560	0.9156
SU-DIPG-XXI	12.15	5.972 to 56.50	0.6026
SU-DIPG-IV	1.475	1.183 to 1.837	0.971
SU-pcGBM2	1.833	1.116 to 3.154	0.871
JHH-DIPG-01	4.379	2.660 to 8.542	0.8091
VUMC-ATRT-03	3.504	2.536 to 4.989	0.916
VUMC-ATRT-01	3.445	2.156 to 6.190	0.8192
CHLA-ATRT-266	75.95	20.31 to 1701	0.7118
VUMC-ATRT-04	2.532	1.520 to 4.443	0.833
VUMC-EPN-06	8.343	6.382 to 11.68	0.8625
VUMC-EPN-05	4.114	2.723 to 6.788	0.8613
VUMC-EPN-01	2.797	2.171 to 3.667	0.9588

a. Effective concentration needed to kill 50% of the cells

Table S2. Significantly correlating Genes

Table S3. Gene Ontology enrichment

Table S4. Known genetic mutations of cell models used in this study

Cell-culture	Entity (Driver mutation)	Other mutations
HSJD-DIPG-07	HGG (H3.3K27M)	CREBBP, MYC, PI3KCA, ACVR1, LRP1B, PPM1B, BRAF, CCND2
VUMC-DIPG-10	HGG (H3WT)	TP53, NF1, MYCN, KRAS, PPM1D
VUMC-DIPGG-G	HGG (H3.3K27M)	TP53, RB1, TERT, PTEN, MGMT, CDKN2A/B
SU-DIPG-XXI	HGG (H3.1K27M)	PDGFRA, ACVR1, MCL1
SU-DIPG-IV	HGG (H3.1K27M)	TP53, PIK3CA, LRP1B, MDM4, ACVR1
SU-pcGBM2	HGG (H3WT)	TP53, NF1, PIK3CA, TERT, RB1
JHH-DIPG-01	HGG (H3.3K27M)	TP53, MYC, MDM4, MET, CDK6, PI3KCA, PTEN
VUMC-ATRT-03	AT/RT-SHH (SMARCB1)	CDKN2A/B, EGFR, CDK6, BRAF, MDM4
VUMC-ATRT-01	AT/RT-SHH (SMARCB1)	MDM4, FGFR3
CHLA-ATRT-266	AT/RT-MYC (SMARCB1)	CREBBP, ARID1A, CDKN2A, FLT4, IGF2
VUMC-ATRT-04	AT/RT-MYC (SMARCB1)	
VUMC-EPN-06	EPN-PFA	
VUMC-EPN-05	EPN-PFA	
VUMC-EPN-01	EPN-PFA	
KNS-42	HGG (H3.3G34)	TP53, PI3KCA, TERT, MLC1
VUMC-DIPG-11	HGG (H3.3K27M)	TP53, MYB, RB1, PDGFRA, CCND1
VUMC-DIPG-F	HGG (H3.3K27M)	MYCN, MET, RB1, PPM1D, BRAF, MDM2
OPBG-GBM-001	HGG (H3.3G34)	TP53, PI3KCA, PDGFRA, BCOR, ATRX

Table S5. Infectious and physical titers of the OVs

OV	physical titer	infectious titer
Delta24-RGD	4.20x10^12 (vp/ml)	9.37 x10^10 (iu/ml)
rQNestin 34.5v1	7.9 x 10^^{11} (GC/ml)	5.2 x 10^9 (PFU/ml)
R124	1.87x10^13 (vp/ml)	1.43 x10^11 (PFU/ml)
rNDV-F0-GFP	N/A	1.6 x10^8 (TCID50/ml)

Table S6. RIN values of RNA samples

RNA sample	RNA Integrity Number (RIN)	rRNA Ratio [28s/18s]
HSJD-DIPG-07	9.7	1.6
VUMC-DIPG-10	9.8	1.9
VUMC-DIPGG-G	9.6	1.6
SU-DIPG-XXI	9.5	1.7
SU-DIPG-IV	9.5	1.7
SU-pcGBM2	9.5	1.6
JHH-DIPG-01	9.9	1.7
VUMC-ATRT-03	9.4	1.7
VUMC-ATRT-01	9.5	1.8
CHLA-ATRT-266	9.5	1.8
VUMC-ATRT-04	9.3	1.5
VUMC-EPN-06	9.8	1.8
VUMC-EPN-05	9.5	1.8
VUMC-EPN-01	9.4	1.6
KNS-42	9.9	2
VUMC-DIPG-11	7.8	1
VUMC-DIPG-F	10	1.7
OPBG-GBM-001	9.4	1.6

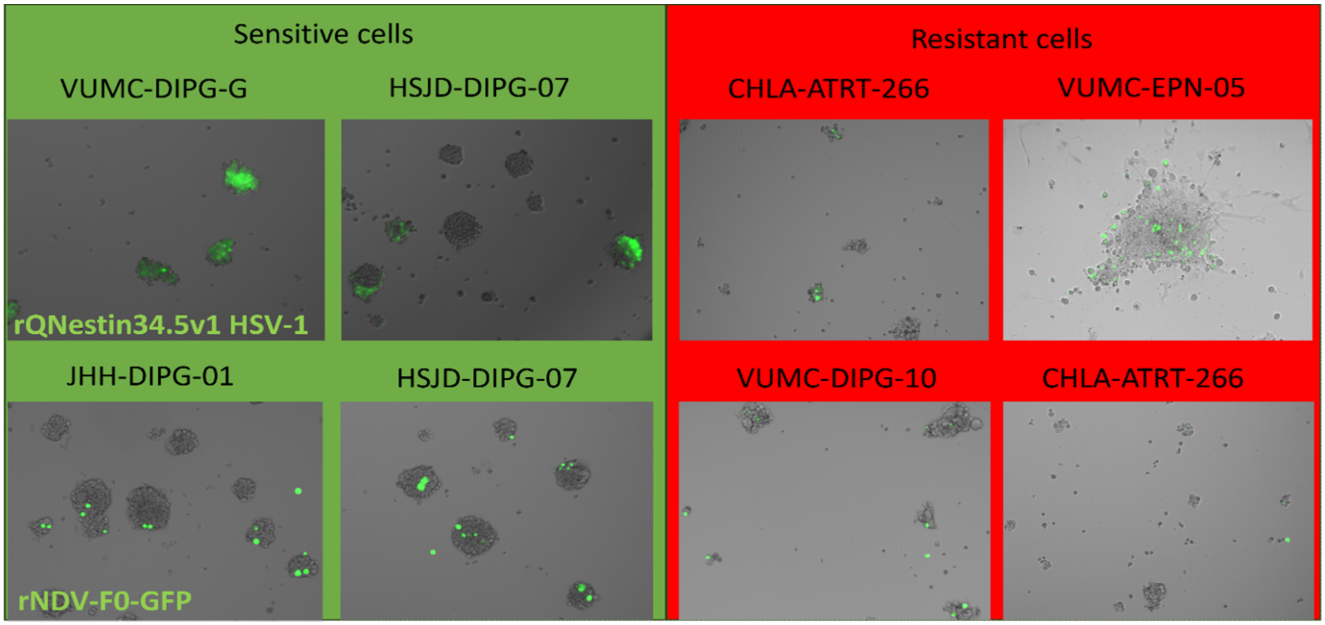

Figure S1. Immunofluorescent depiction of GFP-tagged OV activity.

Immunofluorescent images (20x) of the viral activity of rQNestin34.5v1 and rNDV-F0-GFP five days p.i. with MOI 3.3 and 1.1, respectively, of two representative sensitive PBTs (for rQNestin34.5v1: VUMC-DIPG-G and HSJD-DIPG-07, for rNDV-F0-GFP: JHH-DIPG-01 and HSJD-DIPG-07) and two representative resistant PBTs (for rQNestin34.5v1: CHLA-ATRT-266 and VUMC-EPN-05, for rNDV-F0-GFP: VUMC-DIPG-10 and CHLA-ATRT-266) .
