# Supplementary material for: The heterogeneous sensitivity of pediatric brain tumors to different oncolytic viruses is predicted by unique gene expression profiles
Source: Mol Ther Oncol. 2024 Apr 15;32(2):200804. doi: 10.1016/j.omton.2024.200804 (PMC11060958; doi:10.1016/j.omton.2024.200804)
Supplement: Document S2. Article plus supplemental information [file mmc4.pdf]

Molecular Therapy
Oncology
Original Article

The heterogeneous sensitivity of pediatric brain tumors to different oncolytic viruses is predicted by unique gene expression profiles

Konstantinos Vazaios,[1,2,3] Eftychia Stavrakaki,[2] Lisette B. Vogelezang,[2] Jie Ju,[4] Piotr Waranecki,[1] Dennis S. Metselaar,[1] Michaël H. Meel,[1,5] Vera Kemp,[6] Bernadette G. van den Hoogen,[7] Rob C. Hoeben,[6] E. Antonio Chiocca,[8] William F. Goins,[9] Andrew Stubbs,[4] Yunlei Li,[4] Marta M. Alonso,[10,11,12] Friso G. Calkoen,[1] Esther Hulleman,[1] Jasper van der Lugt,[1,13] and Martine L.M. Lamfers[2,13]

[1]Princess Máxima Center for Pediatric Oncology, Heidelberglaan 25, 3584 CS Utrecht, the Netherlands; [2]Department of Neurosurgery, Brain Tumor Center, Erasmus Medical Center, Dr. Molewaterplein 40, 3015 GD Rotterdam, the Netherlands; [3]Center for Translational Immunology, University Medical Center Utrecht, Heidelberglaan 100, 3584 CX Utrecht, the Netherlands; [4]Department of Pathology and Clinical Bioinformatics, Erasmus Medical Center, Dr. Molewaterplein 40, 3015 GD Rotterdam, the Netherlands; [5]Department of Pediatrics, Wilhelmina Children's Hospital, University Medical Center Utrecht, Lundlaan 6, 3584 EA Utrecht, the Netherlands; [6]Department of Cell and Chemical Biology, Leiden University Medical Center, Einthovenweg 20, 2333 ZC Leiden, the Netherlands; [7]Department of Viroscience, Erasmus Medical Center, Dr. Molewaterplein 40, 3015 GD Rotterdam, the Netherlands; [8]Department of Neurosurgery, Brigham and Women's Hospital, Harvard Medical School, 75 Francis Street, Boston, MA 02115, USA; [9]Department of Microbiology & Molecular Genetics, University of Pittsburgh School of Medicine, 450 Technology Dr, Pittsburgh, PA 15219, USA; [10]Program in Solid Tumors, Center for Applied Medical Research (CIMA), Avda. de Pío XII, 55, 31008 Pamplona, Spain; [11]Department of Pediatrics, Clínica Universidad de Navarra, Av. de Pío XII, 36, 31008 Pamplona, Spain; [12]Health Research Institute of Navarra (IDISNA), Av. de Pío XII, 36, 31008 Pamplona, Spain

Despite decades of research, the prognosis of high-grade pediatric brain tumors (PBTs) remains dismal; however, recent cases of favorable clinical responses were documented in clinical trials using oncolytic viruses (OVs). In the current study, we employed four different species of OVs: adenovirus Delta24-RGD, herpes simplex virus rQNestin34.5v1, reovirus R124, and the non-virulent Newcastle disease virus rNDV-F0-GFP against three entities of PBTs (high-grade gliomas, atypical teratoid/rhabdoid tumors, and ependymomas) to determine their *in vitro* efficacy. These four OVs were screened on 14 patient-derived PBT cell cultures and the degree of oncolysis was assessed using an ATP-based assay. Subsequently, the observed viral efficacies were correlated to whole transcriptome data and Gene Ontology analysis was performed. Although no significant tumor type-specific OV efficacy was observed, the analysis revealed the intrinsic biological processes that associated with OV efficacy. The predictive power of the identified expression profiles was further validated *in vitro* by screening additional PBTs. In summary, our results demonstrate OV susceptibility of multiple patient-derived PBT entities and the ability to predict *in vitro* responses to OVs using unique expression profiles. Such profiles may hold promise for future OV preselection with effective oncolytic potency in a specific tumor, therewith potentially improving OV responses.

INTRODUCTION

Pediatric central nervous system (CNS) tumors are the leading cause of death by disease for age groups 0 to 18.[1] High-grade gliomas (HGGs) have very poor survival and even in children with gross total resection, the 5-year survival rate is below 20%.[2] Specifically, the Histone 3 (H3K27M)-altered diffuse midline gliomas (DMGs) carry a median survival of 11 months, largely because they are irresectable.[3] Atypical teratoid/rhabdoid tumors (AT/RTs) are brain tumors of embryonal origin that account for 1% to 2% of all pediatric CNS tumors in children <3 years old.[4] Overall, AT/RTs have poor prognosis with 5-year survival rate ≤28%, especially in individuals younger than 3.[5] Ependymomas (EPNs) are the third most common CNS tumor in children accounting for approximately 10% of cases.[6] DNA methylation profiling has divided the aforementioned entities into several subgroups each with a preferable anatomic compartment, age of presentation, malignancy grade, and prognosis tightly linked to those groups.[7–10] Briefly, according to the Brain classifier version 12.8 (https://www.molecularneuropathology.org/mnp/classifiers/14) from Heidelberg, HGGs are divided into H3 wild type (WT) and IDH WT, H3 G34-mutant, MYCN, RTK1, RTK2, DMG H3K27 and epidermal growth factor receptor (EGFR), DMG H3K27/EZHIP subtypes; AT/RTs are divided into MYC, SHH ,and TYR subtypes; and EPNs are divided into Myxopapillary, Posterior fossa group A (PFA)1a-f, PFA2a-c, PFB1-5, spinal, spinal and MYCN-amplified,

Received 11 October 2023; accepted 11 April 2024;
https://doi.org/10.1016/j.omton.2024.200804.

[13]These authors contributed equally

Correspondence: Jasper van der Lugt, MD, PhD, Princess Máxima Center for Pediatric Oncology, Heidelberglaan 25, 3585 CS Utrecht, the Netherlands.
E-mail: j.vanderlugt@prinsesmaximacentrum.nl

This is an open access article under the CC BY license (http://creativecommons.org/licenses/by/4.0/).

Table 1. Oncolytic virus entry-related genes mentioned in publications

Oncolytic virus	Viral entry-related genes
Delta24-RGD	IntegrinαVβ5 (*ITGA5*), IntegrinαVβ (*ITGAV*), IntegrinαVβ3 (*ITGA3*), Coxsackie Adenovirus Receptor (*CAR;CXADR*)[42]
rQNestin34.5v1	Herpesvirus Entry Mediator (*HVEM;TNFRSF14*), Syndecan-1 (*SDC1*), Syndecan-2 (*SDC2*), *NECTIN 1, NECTIN 2*, Paired Immunoglobin Like Type 2 Receptor Alpha (*PILRA*), Non-muscle Myosin Heavy Chain IIA (*NMHC-IIA;MYH9*), Perlecan (*HSPG2*)[43–46]
R124	Junction Adhesion Molecule A (*JAM-A;F11R*), Epidermal Growth Factor Receptor (*EGFR*), Nogo Receptor (*NgR1;RTN4R*)[26,30,47,48]
rNDV-F0-GFP	Cavin-1 (*CAVIN1*), Ras related protein AB5 (*RAB5*), Caveolin-1 (*CAV1*)[49,50]

Spinal subEPN A and B, supratentorial EPN YAP1-fused, ZFTA fused, ZFTA::RELA fused, and supratentorial subEPN.[10]

The standard treatment for high-grade PBTs consists of a combination of surgical resection, when feasible, followed by chemotherapy and/or radiotherapy. Over the past decades, little improvement in survival has been made compared with other pediatric tumors. Moreover, treatment comes with long-term morbidity, resulting in a significant decline in quality of life.[11] Therefore, there is an urgent need for alternative and less invasive treatment strategies to reduce the long-term treatment effects, while improving survival rates.

An upcoming alternative option that could help overcome these challenges is the use of oncolytic viruses (OVs). OVs specifically infect tumor cells, replicate, and spread to neighboring uninfected tumor cells, causing tumor cell lysis.[12–14] In addition, the oncolytic activity of the OV can trigger a local inflammatory response through the release of pathogen-associated molecular patterns (PAMPS), damage-associated molecular patterns (DAMPS), cytokines, and chemokines. This response, along with the release of tumor-associated antigens (TAAs) from infected tumor cells, subsequently initiates a systemic anti-tumor immune response.[12,15–17] Numerous DNA and RNA virus species, such as adenovirus (AdV), herpes simplex virus (HSV), reovirus (RV), and Newcastle disease virus (NDV), have been tested in preclinical models and have been studied in clinical trials against PBTs with various successes.[18–21] More specifically, Delta24-RGD, also known as DNX-2401, is a type 5 human AdV genetically modified with a 24-base pair deletion on its *E1A* gene and an addition of an arginine-glycine-aspartic acid (RGD) motif on its fiber protein.[22] These modifications enable the virus to interact with α_v integrins for attachment and entry and to replicate in cells with a dysfunctional retinoblastoma tumor suppressor (RB) pathway, thereby providing tumor cell specificity.[22] Promising clinical results were obtained during a recent clinical trial, where DNX-2401 was administered intratumorally in 12 DMG patients, which led to a median overall survival of 17.8 months compared with the historic average of 11 months.[18] rQNestin34.5v1 is a neurotropic HSV-1 modified to target cells overexpressing Nestin, such as glioblastoma and cancer stem-like cells, and not the surrounding healthy cells, thereby providing tumor selectivity.[23–25] R124 is an unmodified human RV type 3 Dearing (T3D), which has been reported to have tropism toward cells with an upregulated Ras/RalGEF/p53 pathway.[19,26–29] This virus displayed highly variable cytotoxicity in a panel of glioblastoma cell lines.[30] Finally, rNDV-F0-GFP is an avirulent NDV strain La Sota with a mono-basic cleavage site in the fusion protein (F0) for increased virulence, NDVs often, but not consistently, demonstrate a natural preference for cells with deficiencies in the type I interferon (IFN) pathway, as reported for numerous cancer cell types including adult glioblastoma.[21,31–34]

Despite an increasing number of viruses being investigated for clinical use, no studies have covered the oncolytic potential of those viruses against an extended number of PBTs. Recent, *in vitro* studies have employed patient-derived monocultures of brain tumor spheres that retain the phenotypic and molecular profile of the original tumor.[35,36] Compared with the classical monolayers, tumor cell-derived spheres are able to recapitulate cell-cell, cell-ECM interactions, as well as quiescent and proliferative zones similar to *in vivo* tumors.[36,37] Studies on screening of patient-derived tumor spheres and/or spheroids have also demonstrated small molecule drug sensitivities resembling the *in vivo* efficacy or enabled responder identification, stratification to new treatments, or potential combination synergies.[38–41] Therefore, applying patient-derived *in vitro* models may provide a suitable platform for preclinical evaluation of OVs and discovery of predictive factors for OV oncolytic efficacy.

In this study, we evaluated the oncolytic potential of four OVs, Delta24-RGD, rQNestin34.5v1, R124, and rNDV-F0-GFP, on a panel of patient-derived tumor sphere cultures covering a range of PBT entities. Our findings highlight the oncolytic potential of these OVs against PBTs and shed light on the molecular factors contributing to the heterogeneous response observed for each virus. By correlating genes and biological processes with the specific sensitivity and resistance to the OVs, we take a first step toward identifying predictive molecular tumor signatures in OV therapy.

RESULTS

PBTs express genes related to viral entry for the four OVs

We performed *in silico* analysis of the transcriptome of patient-derived PBT sphere cultures (n = 14) belonging to the entities HGG (n = 7), AT/RT (n = 4), and EPN (n = 3). Assessment of RNA levels of genes related to viral cell attachment and entry for the four OVs, as summarized in Table 1, revealed that most viral entry-related genes were expressed by the cell cultures to some degree (Figure 1), with the exception of *HVEM, PILRA*, and *F11R*, which were below detection levels (data not shown). These genes covered entry-related molecules for each of the four OVs, indicating that the selected OVs would be expected to infect patient-derived HGGs, AT/RTs, and EPNs (Figure 1).

Oncolytic effect of the OVs in patient-derived PBT cultures

Having demonstrated the expression of genes that serve as entry-related molecules for the four OVs, we screened dose ranges of Delta24-RGD,

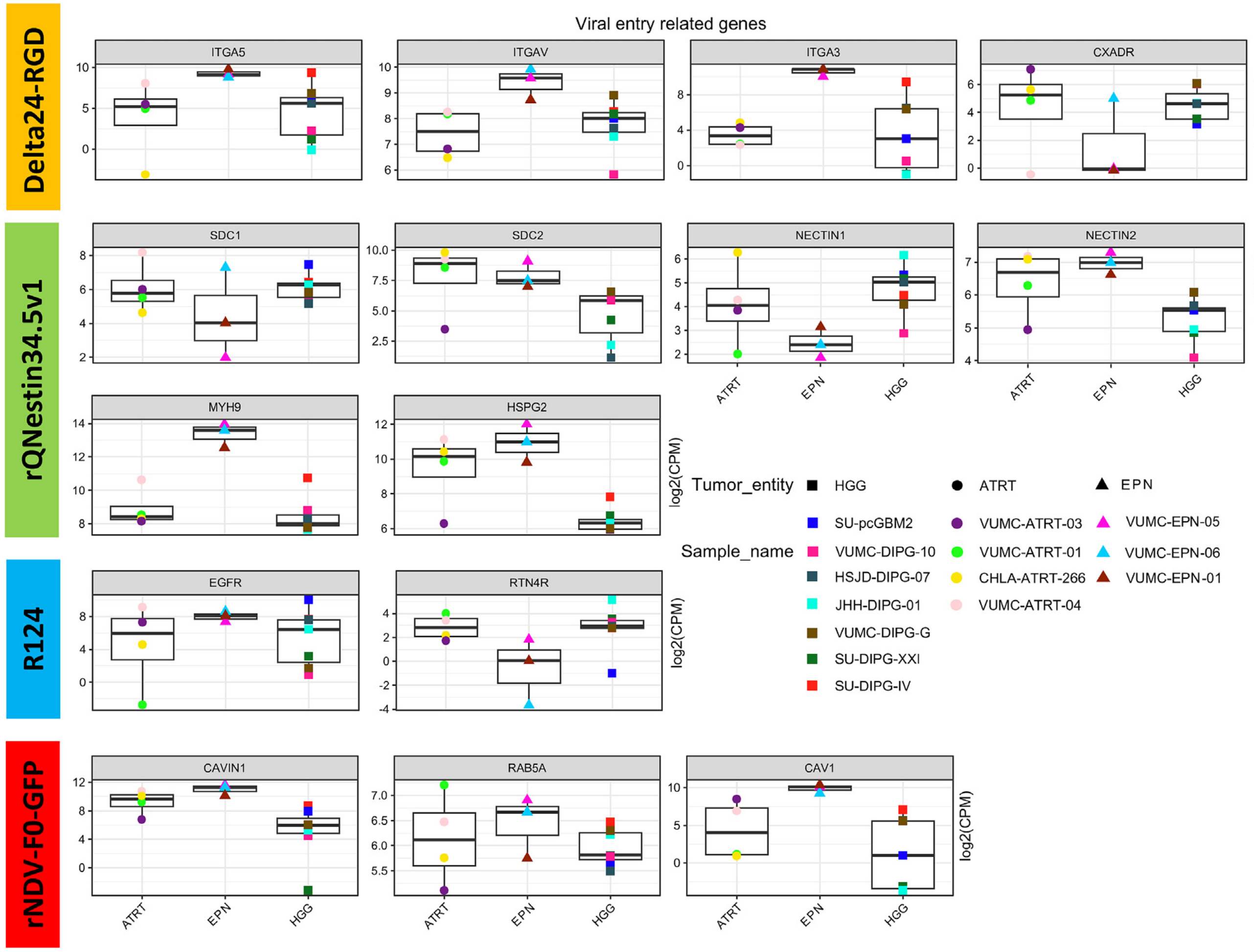

Figure 1. Delta24-RGD, rQNestin34.5v1, R124, and rNDV-F0-GFP entry-related genes in HGG, AT/RT, and EPN

Every point represents a unique patient-derived cell culture ($n = 14$) with every sign being a cell culture of specific tumor entity ($n = 3$). Each boxplot corresponds to the median expression of one gene per tumor entity. The y axis represents the transcription levels of the selected genes on a free scale after log2 transformation of the normalized counts per million (CPM), while the x axis represents the tumor entities in which the cell cultures were grouped.

rQNestin34.5v1, R124, and rNDV-F0-GFP on the PBTs. As depicted in Figure 2A, large variability in response was observed to each OV by each tumor type. To compare the efficacy of the four OVs over the panel of PBTs, we calculated the effective concentration for 50% oncolysis (EC_{50}) per OV (Figure 2B; Table S1). As the OVs are each effective within different dose ranges, we only compared the EC_{50} values across PBTs and not across OVs. Mean EC_{50} values for Delta24-RGD ranged from multiplicity of infection (MOI) 2.6 to >50 in HGGs, 4.5 to 9.5 in AT/RTs, and 6.6 to 14 in EPNs (Figure 2B). rQNestin34.5v1 ranged from MOI 0.1 to 0.8 in HGGs, 0.1 to >30 in AT/RTs and 0.4 to >30 in EPNs. R124 ranged from MOI 0.1 to >300 in HGGs, 0.3 to >300 in AT/RTs, and 197.3 to >300 in EPNs. rNDV-F0-GFP ranged from MOI 1.5 to >10 in HGGs, 2.5 to >10 in AT/RTs, and 2.8 to 8.3 in EPNs (Figure 2B). Despite this great variability in OV susceptibility over the PBT panel, every PBT cell culture demonstrated sensitivity to at least one OV, with no significant tumor entity preference noted for any individual OV (Figures 2B and 2C). In summary, these results demonstrate that Delta24-RGD, rQNestin34.5v1, R124, and rNDV-F0-GFP can induce a significant and heterogeneous oncolytic effect in most of the tested PBT cultures. Representative immunofluorescent images of two sensitive and two resistant PBTs on day 5 post infection are depicted as examples of the viral activity of rQNestin34.5v1 and rNDV-F0-GFP in the PBT spheres (Figure S1).

Correlating OV sensitivity and resistance to basal gene expression

To identify genes associated with sensitivity and resistance to the OVs, we performed bulk RNA-sequencing to obtain the transcriptional profiles of these 14 highly heterogeneous PBT sphere cultures in their uninfected state. Using the OVs EC_{50} for the 14 cultures, we assessed Spearman's correlation coefficient (ρ) and p values. A ρ value lower than -0.5 with a p value of <0.05 indicated a gene that

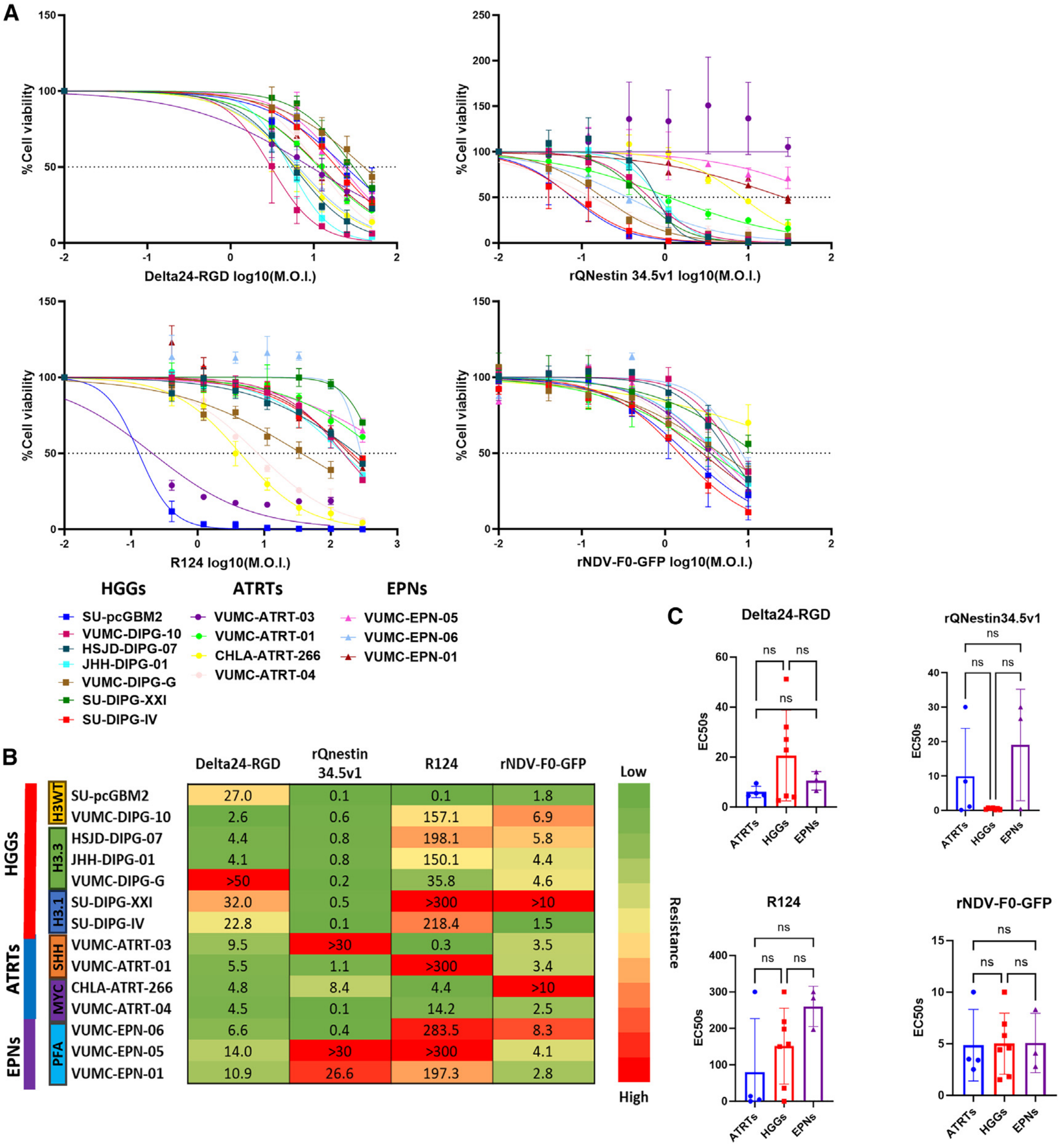

		Delta24-RGD	rQnestin 34.5v1	R124	rNDV-F0-GFP
HGGs (H3WT)	SU-pcGBM2	27.0	0.1	0.1	1.8
HGGs (H3.3)	VUMC-DIPG-10	2.6	0.6	157.1	6.9
HGGs (H3.3)	HSJD-DIPG-07	4.4	0.8	198.1	5.8
HGGs (H3.3)	JHH-DIPG-01	4.1	0.8	150.1	4.4
HGGs (H3.3)	VUMC-DIPG-G	>50	0.2	35.8	4.6
HGGs (H3.1)	SU-DIPG-XXI	32.0	0.5	>300	>10
HGGs (H3.1)	SU-DIPG-IV	22.8	0.1	218.4	1.5
ATRTs (SHH)	VUMC-ATRT-03	9.5	>30	0.3	3.5
ATRTs (SHH)	VUMC-ATRT-01	5.5	1.1	>300	3.4
ATRTs (MYC)	CHLA-ATRT-266	4.8	8.4	4.4	>10
ATRTs (MYC)	VUMC-ATRT-04	4.5	0.1	14.2	2.5
EPNs (PFA)	VUMC-EPN-06	6.6	0.4	283.5	8.3
EPNs (PFA)	VUMC-EPN-05	14.0	>30	>300	4.1
EPNs (PFA)	VUMC-EPN-01	10.9	26.6	197.3	2.8

Figure 2. Cell viability assays after OV infection

(A) Patient-derived cell cultures were incubated with various dilutions multiplicities of infection (M.O.I.) of Delta24-RGD, rQNestin34.5v1, R124, and rNDV-F0-GFP and cell viability was measured 5 days post OV infection with CTG assay. The data are shown as the percentage of cells alive after infection with Delta24-RGD, rQNestin34.5v1, R124, or rNDV-F0-GFP at the indicated log10 (M.O.I.s) relative to the non-infected cells, with each point corresponding to the mean ± (SD) values of independent CTG assays per cell line (*n* = 2 to 4) (see also Table S1). (B) EC_{50} obtained from (A), the color scaling is independent per OV from green (lowest EC_{50}) to red (highest EC_{50}). (C) Mean EC_{50} comparisons grouped per tumor entity. Each point in the bar plots corresponds to the EC_{50} of a specific PBT cell culture. For the comparison of the EC50 per tumor entity the non-parametric Kruskal-Wallis test with Dunn's multiple comparison correction. ns = not statistically significant.

significantly correlated with sensitivity to the OV, while a ρ value higher than 0.5 with a *p* value <0.05 showed that a gene was associated with resistance (Table S2). Unexpectedly, we found that none of the virus entry-related genes showed a significant correlation with sensitivity to the OVs, except for syndecan 1 (*SDC1*) with $\rho < -0.79$ (*p* value <0.0007) for rQNestin34.5v1 (Figures 3A–3D).

The number of genes correlated with sensitivity and resistance, respectively, varied greatly among the different OVs (Figures 3A–3D; Table S2). The 10 most correlating genes for Delta24-RGD, rQNestin 34.5v1, R124, and rNDV-F0-GFP are shown in Figures 3A–3D. Interestingly, we observed no overlap between the genes associated with sensitivity and resistance to each

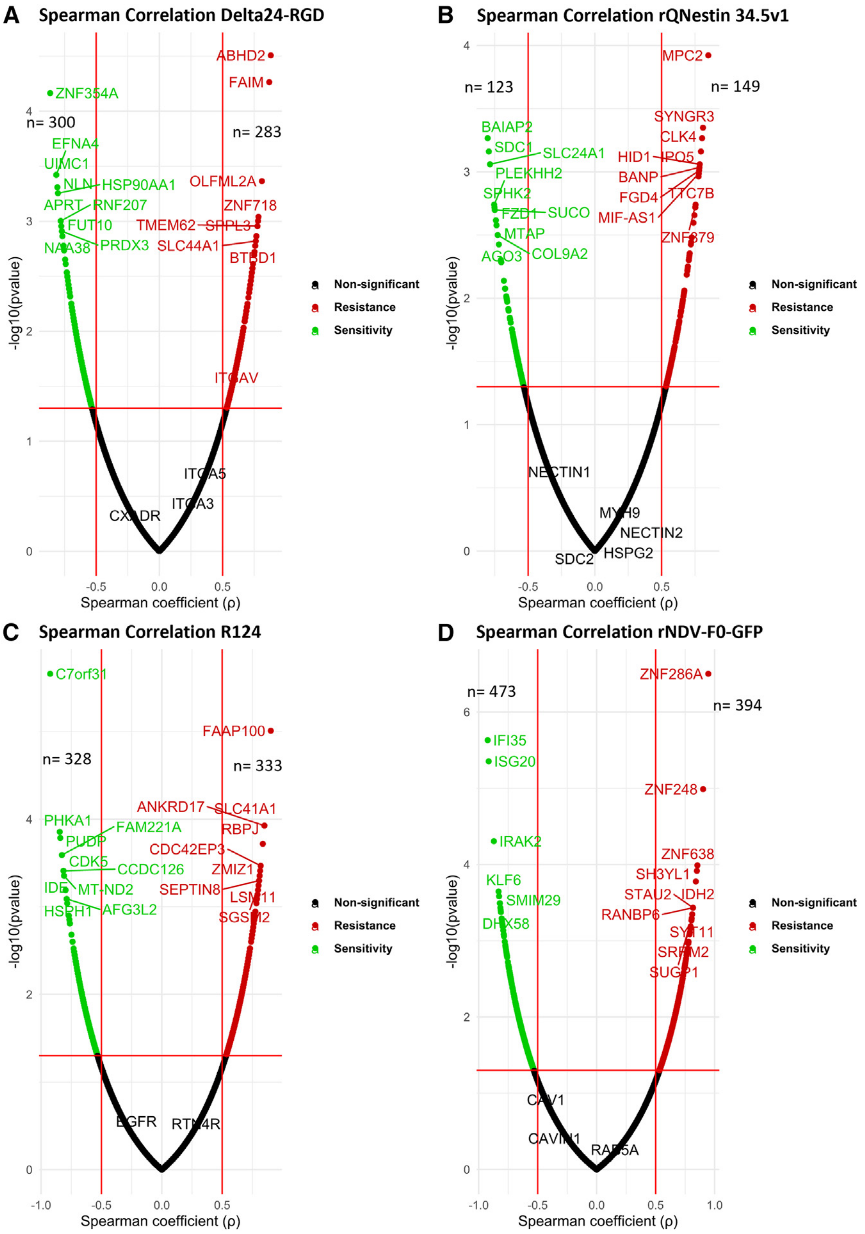

Figure 3. Correlating genes of OV sensitivity and resistance

Volcano plots of Spearman's correlation coefficient (ρ) values demonstrating significantly correlated genes for sensitivity and resistance for (A) Delta24-RGD, (B) rQNestin34.5v1, (C) R124, and (D) rNDV-F0-GFP, resulting from correlating gene expression and EC_{50} values (see also Table S2). The x axis represents the Spearman's correlation coefficient (ρ), and the y axis represents the −log10 (*p* value). Each dot represents a gene, with black the non-significant (absolute coefficient <0.5 and *p* value <0.05), green the sensitivity-related genes (coefficient < −0.5 and *p* value <0.05), while red the resistance-related genes (coefficient >0.5 and *p* value <0.05). Labeled are the genes related to viral entry using their gene symbol. Volcano plots were created with R (https://www.r-project.org/). Spearman correlation was employed.

OV, suggesting distinct molecular mechanisms underlying these responses.

Gene Ontology term enrichment of OV correlated genes

To characterize the biological processes associated with sensitivity and resistance to the different oncolytic viruses, we analyzed the sets of genes that showed significant correlations with sensitivity and resistance (Table S2). We performed Gene Ontology (GO) term enrichment analysis to identify distinct biological processes linked to susceptibility to each specific OV (Figures 4A–4D; Table S3). For Delta24-RGD sensitivity, the related genes were enriched in terms associated with viral transcription and viral gene expression (Figure 4A). Resistance-related genes were enriched in terms related to NF-κB signaling and vesicle organization (Figure 4A). The sensitivity to rQNestin34.5v1 was associated with cellular growth, WNT signaling, and glycoprotein synthesis, whereas resistance was related to cerebellum vasculature development and ribosomal protein import into the nucleus (Figure 4B). Genes associated with R124 sensitivity were enriched in terms related to mitochondrial activity and RNA processing, R124 resistance was linked to terms such as positive GTPase regulation, autophagy, and Ras protein signaling (Figure 4C). rNDV-F0-GFP sensitivity-related genes were enriched in terms related to NF-κB signaling, glycoproteins, and adhesion (Figure 4D). Conversely, resistance to rNDV-F0-GFP was linked to terms related to transcription and translation (Figure 4D).

Validation of the molecular profiles of sensitivity and resistance

Correlation of EC_{50} values with RNA expression of the PBT cultures before infection provided us with a list of genes that enrich for biological functions relating to each OV. To assess the potential of the correlated genes for sensitivity and resistance to predict OV cytotoxic potential, we extended our screening with four additional PBT sphere cultures (KNS-42, OPBG-GBM-001, VUMC-DIPG-11, VUMC-DIPG-F), referred to as validation cultures. Their molecular signatures were compared with previously utilized PBT cultures, referred to as confirmation cultures, by unsupervised clustering using the gene expression profiles for sensitivity and resistance (Table S2) and by *in vitro* OV sensitivity testing.

Based on the signature for Delta24-RGD sensitivity, the molecular profiles of the four PBT validation cultures clearly clustered with the

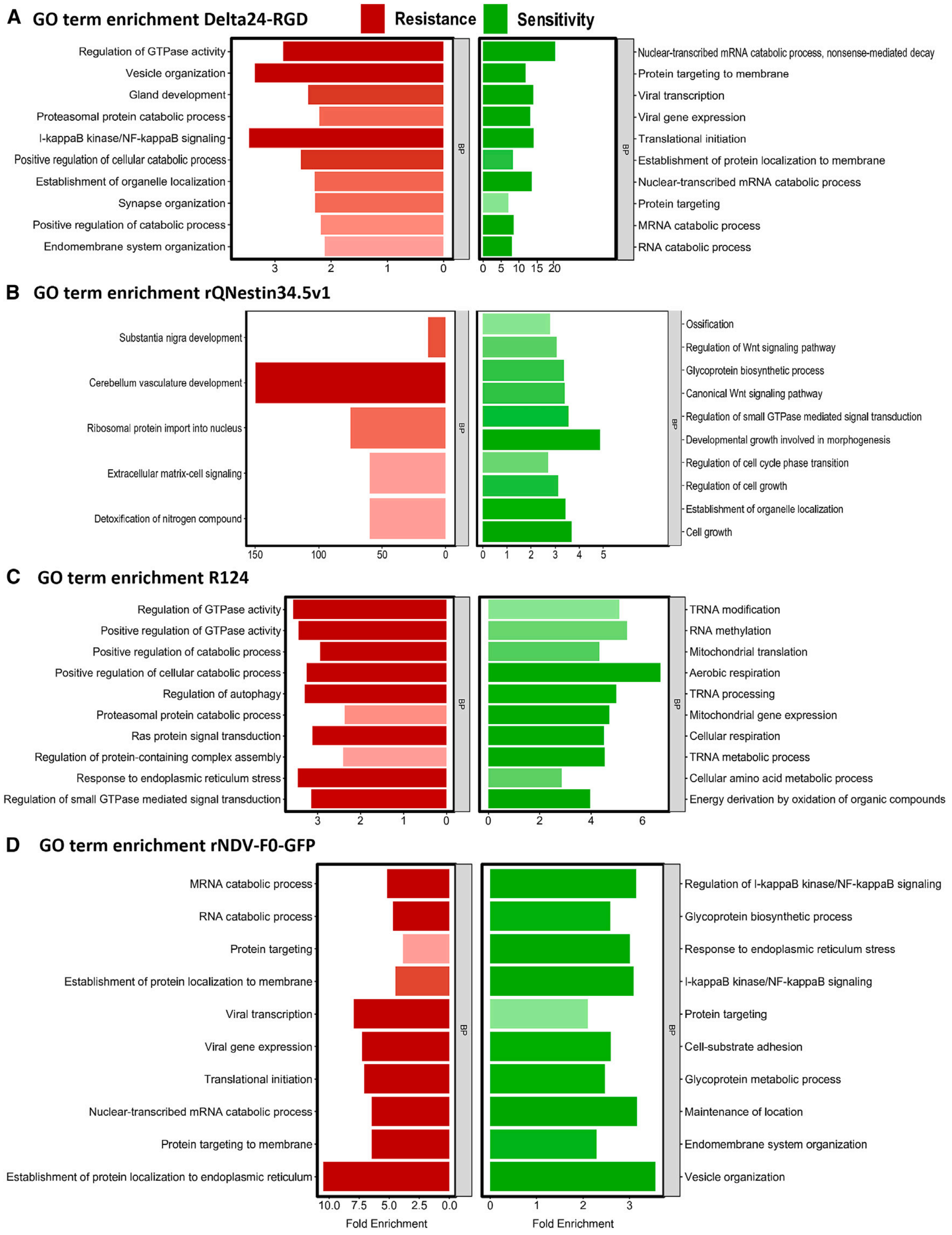

Figure 4. Top 10 most enriched biological processes terms of Gene Ontology

The bar plots demonstrate the top 10 Gene Ontology biological processes terms based on the numbers of genes enriched after GO enrichment analysis for Delta24-RGD (A), rQNestin34.5v1 (B), R124 (C), and rNDV-F0-GFP (D). The bar plots are color-coded with green being related to sensitivity and red related to resistance. Significant GO terms (*p* value <0.05) are depicted (see also Table S3). The terms with the lowest *p*-adjusted value are demonstrated with a darker shade of red or green while terms with higher *p* value are depicted with lighter shades of red or green, respectively. Bar plots were created with R (https://www.r-project.org/). The *p*-adjusted value was calculated through the FDR method.

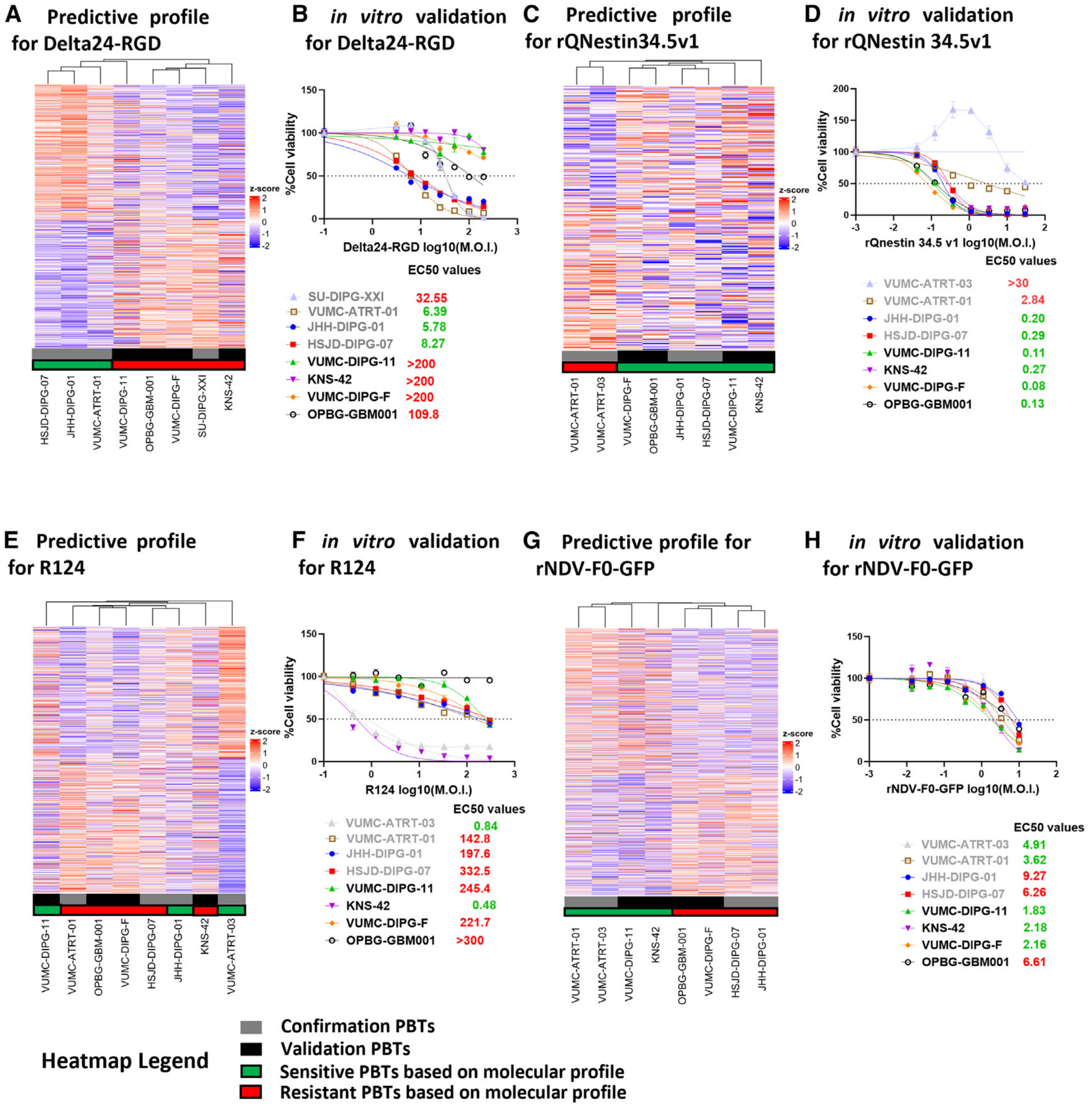

Figure 5. *In vitro* validation of OV sensitivity signatures

Each heatmap represents the expression of genes that significantly correlated with Delta24-RGD (A), rQNestin34.5v1 (C), R124 (E), and rNDV-F0-GFP (G) resistance and sensitivity. Each column represents a PBT culture cluster based on the average expression of the genes. Each line represents the Z score expression levels of all correlated genes for resistance and sensitivity. Cell viability graphs of four confirmation PBT cultures in gray and four newly tested validation PBT cultures in black after 5-day incubation with Delta24-RGD (B), rQNestin34.5v1 (D), R124 (F), and rNDV-F0-GFP (H). Cell viability was measured for the validation of the predictive profiles as described in Figure 2A. Each point represents the mean ± (SD) (n = 3). Heatmaps were plotted in R (https://www.r-project.org/).

resistant confirmation culture SU-DIPG-XXI, while clustering the sensitive confirmation cultures JHH-DIPG-01, HSJD-DIPG-07, and VUMC-ATRT-01 together, thus predicting the resistance of all validation cultures (Figure 5A). Indeed, upon *in vitro* testing for Delta24-RGD, the four validation cultures proved to be highly resistant, with EC_{50} values higher than 50 (Figure 5B). For rQNestin34.5v1, the molecular signatures of all four validation cultures clustered together with those of the sensitive confirmation cultures JHH-DIPG-01 and HSJD-DIPG-07, while the resistant confirmation cultures VUMC-ATRT-03 and VUMC-ATRT-01 clustered separately (Figure 5C). Again, dose-response testing with rQNestin34.5v1 resulted in EC_{50} values lower than 0.3 in all four validation cultures, in accordance with predicted sensitivity (Figure 5D). In the case of R124, the unsupervised clustering of the molecular signatures did not fully separate the sensitive from the resistant cultures (Figure 5E). Of the three predicted resistant validation cultures OPBG-GBM-001, VUMC-DIPG-F, and KNS-42, the latter revealed a highly sensitive *in vitro* response to R124 with an EC_{50} of 0.48. Inversely, the validation cultures VUMC-DIPG-11 that demonstrated a sensitive profile revealed an EC_{50} value of 221.7 (Figure 5F). The remainder of the validation cultures (VUMC-DIPG-F and

OPBG-GBM-001) clustered with the resistant VUMC-ATRT-01 and HSJD-DIPG-07 (Figure 5E) and *in vitro* validation indeed demonstrated resistance to R124 (Figure 5F). Finally, the molecular profiles for rNDV-F0-GFP also resulted in two clear clusters, delineating predicted sensitive and resistant PBT cultures to rNDV-F0-GFP (Figure 5G). *In vitro* validation showed that the expression profiles successfully predicted the relative sensitivity of the validation cultures KNS-42 (EC_{50} = 2.18), VUMC-DIPG-11 (EC_{50} = 1.8), as well as the resistance of OPBG-GBM-001 (EC_{50} = 6.61), while failing to predict the sensitivity of VUMC-DIPG-F with EC_{50} = 2.16 (Figure 5H). Taken together, the analysis shows that the identified signatures of OV sensitivity can act as biomarkers for predicting the relative sensitivity or resistance of PBT cell cultures to OV-induced oncolysis, in particular for Delta24-RGD and rQnestin34.5v1.

DISCUSSION

Several OVs have been investigated both in preclinical and clinical studies for treatment of PBTs, showing a beneficial safety profile and promising efficacy, either as monotherapy or when combined with radiotherapy or chemotherapy.[51,52] Ongoing clinical investigations using OVs in PBTs remain limited to medulloblastoma and HGG (AdVs: NCT03178032, NCT05717712, NCT05717699, NCT 0458533. HSVs: NCT02457845, NCT03911388, NCT04482933, NCT02031965. RVs:NCT02444546. NDVs: NCT01174537. Measles viruses: NCT02962167. PVs: NCT03043391), while in preclinical investigations of DNX-2401 AT/RTs were included.[19,42,51,53–55] No prior study has included EPNs with OVs. Here, we assessed the oncolytic efficacy of Delta24-RGD, rQNestin34.5v1, R124, and rNDV-F0-GFP for HGGs, AT/RTs, and EPNs.

Our investigation demonstrated the expression of viral entry genes for the different OVs by each tumor entity and the effective oncolysis of PBTs. The use of Delta24-RGD on HGGs and AT/RTs resulted in EC_{50} ranges similar to those previously reported by Alonso et al.,[42,53] demonstrating the reproducibility of our screening process. Interestingly, Delta24-RGD showed high oncolytic potency against many AT/RTs and EPNs with MOIs below 10. HGGs also demonstrated a general sensitivity to rQNestin34.5v1 with MOIs below 1. However, when comparing the groups, none of the OVs displayed significant oncolytic potency to any individual PBT entity.

The use of patient-derived tumor sphere cultures has become the gold standard in drug screening and has contributed to the identification of cell-intrinsic factors that affect therapeutic efficacy, while better reflecting clinical responses.[38,41,56,57] As we observed a considerable variability in EC_{50} ranges across PBTs, even within the same tumor entity, we decided to capitalize on tumor cell-intrinsic factors that dictate sensitivity or resistance to each OV by correlating the transcriptome of the PBT cultures with their EC_{50} values. As each OV employs different mechanisms in the host cells to induce its cytotoxic action, this approach could potentially yield biomarkers of response. Indeed, a recent systematic analysis of all publications relating to oncolytic virus resistance demonstrated that the main source of resistance to OV therapy originates from different cell-intrinsic factors, including cell survival, heterogeneity, hypoxia, growth factors, epigenetic factors, viral entry, and interferon responses.[58]

Our analysis provided a list of genes correlating either with sensitivity or resistance to each OV. Interestingly, of the 18 viral entry-related genes, only the expression of the HSV entry receptor *SDC1*, which was previously shown to greatly influence the spread of HSV-1,[59] significantly correlated with sensitivity. The relevance of the correlated genes in our study became more apparent when they were mapped to GO biological processes relevant to each OV. The GO terms for Delta24-RGD align with the well-known importance of the interplay between transcription, translation, and AdV-host interactions across different timepoints of AdV5 infection, early points of infection have demonstrated to be heavily dependent on chromatin acetylation.[60–63] Splicing-related terms were also correlated with sensitivity, a process first discovered in AdVs and essential for infection cycle.[64] In the case of resistance, terms related to NF-κB regulation were also consistent with previous studies indicating that inhibition of NF-κB enhances the oncolytic cytotoxicity of AdVs.[65] Terms related to hypoxia together with increased expression of *HIF-1A* correlated with Delta24-RGD resistance, a process known to negatively affect the cytolytic effect and production of AdV5 without affecting viral entry in all cells tested.[66] In addition to these terms, the gene *Mx1(MxA)* correlated with Dela24-RGD resistance, as previously reported in adenovirus resistance.[67]

Glycoproteins are essential for HSV entry and GO terms as "glycoprotein biosynthetic process," "protein glycosylation," and "aminoglycan biosynthesis" were identified, thus highlighting their importance as a cell-intrinsic factor affecting PBT sensitivity to rQNestin34.5v1.[43,68,69] Furthermore, the term "exosomal secretion" was also related to sensitivity. Exosomes have been demonstrated as an important tool for HSV-1 release from infected cells and spreading to uninfected cells.[70] WNT/β-catenin signaling has been associated with HSV-1 productive infection and our analysis also associated to this pathway with PBT sensitivity to rQNestin34.5v1.[71]

The dependence of R124 to both GTPase activity and Ras pathway signaling was clearly demonstrated in GO terms like "negative regulation of Ras protein signaling" and "positive regulation of GTPase activity," which correlated with PBT resistance to R124.[19,26–28] Briefly, GTPase activity has been reported to downregulate the Ras protein superfamily, as it hydrolyses the active RAS-GTP to its inactive form, while Ras over-activation is crucial for promoting RV activity.[26–28,72,73] In addition, the analysis revealed the relevance of mitochondrial activity in the context of RV, which aligns with its reported importance in inducing apoptosis upon RV infection.[74]

In the case of rNDV-F0-GFP, sensitivity GO terms such as "vesicle organization," "vesicle coating," "regulation of endocytosis," and "endosome to lysosome transport" highlight the importance of the different entry mechanisms as a factor of successful oncolysis. The ability of NDV to utilize multiple endocytosis pathways for cell entry have previously been demonstrated.[49,75,76] Recent observations have

indicated that NF-κB-triggered JNK activation promotes apoptosis and inflammation, supporting rNDV-F0-GFP proliferation and validating the GO terms "IRE1-mediated unfolded protein response" and "I-kappaB kinase/NF-κB signaling."[77–79] In addition to those terms, the gene *ATG5* significantly correlated with PBT sensitivity to rNDV-F0-GFP, aligning with previously reported siRNA silencing of *ATG5* affecting NDV production in U251 glioma cells.[80]

Overall, our analysis uncovered a number of genes and GO terms of importance in different stages of the infection cycles of the OV species applied. These findings provide insights into the underlying mechanisms that affect oncolytic efficacy of OVs in pediatric brain tumors (PBTs).

Importantly, the predictive value of the correlated genes was validated in an independent set of PBT cultures with the expression profiles for Delta24-RGD and rQNestin34.5v1, correctly clustering and predicting the relative sensitivity or resistance of all four new cultures. In addition, the markers for rNDV-F0-GFP were able to correctly predict sensitivity of three of the four newly tested PBT cultures. For R124, the signature genes were not able to predict as effectively the relative resistance/sensitivity of the newly tested cultures.

Validation of the correlated genes as biomarkers for sensitivity or resistance to specific OVs *in vitro*, represents a first step toward a tailored OV therapy. However, it is essential to evaluate the predictive value of the identified molecular profiles on a larger, independent cohort of pediatric brain tumor samples. Moreover, our *in vitro* model system does not support assessment of the other key mode of action of OVs, namely generation of an anti-tumor immune response. More advanced *ex vivo* models, incorporating the tumor microenvironment and/or immune components, such as autologous tumor/PBMC cocultures, may aid in acquiring further insight into the relationship between OV-induced oncolysis and degree of immune stimulation.[81] Furthermore, concurrently screening of patient-derived tumor samples alongside clinical investigations may assist in the further validation and refinement of the identified molecular signatures.

In conclusion, our study demonstrates that Delta24-RGD, rQNestin34.5v1, R124, and rNDV-F0-GFP hold oncolytic potential for multiple entities of PBTs. By screening multiple patient-derived cell cultures, we were able to identify cell-intrinsic factors that relate to sensitivity and resistance to the four selected OVs. In addition, the predictive power to oncolytic efficacy *in vitro* of those genes was validated. As we continue to explore and validate such biomarkers in future translational research, we move closer to developing personalized medicine and improving the efficacy of oncolytic virus-based therapies for PBT patients.

MATERIALS AND METHODS

Patient-derived cell cultures

HSJD-DIPG-07 (H3.3K27M, DMG) was a kind gift from Dr. Carcaboso (Hospital Sant Joan de Déu, Barcelona, Spain)[82]; JHH-DIPG-01 (H3.3K27M, DMG) were provided by Dr. Raabe (John Hopkins Hospital, Baltimore, MD, USA)[83]; SU-DIPG-IV (H3.1K27M, DMG), SU-DIPG-XXI (H3.1K27M, DMG), and SU-pcGBM2 (H3WT, GBM) were provided by Dr. Monje (Stanford University, Stanford, CA, USA)[84]; CHLA-ATRT-266 (MYC, ATRT) was obtained from the American Type Culture Collection (ATCC)[85]; KNS-42 (H3.3G34, DMG) was obtained from the JCRB (Japan Cancer Research Resources) cell bank[86]; OPBG-GBM-001 (H3WT, GBM) was provided by Dr. Vinci (Bambino Gesú Children's Hospital); while VUMC-DIPG-10 (H3WT, DMG), VUMC-DIPG-G (H3.3K27M, DMG), VUMC-DIPG-11 (H3.3K27M, DMG), VUMC-DIPG-F (H3.3K27M, DMG), VUMC-ATRT-03 (SHH, ATRT), VUMC-ATRT-01 (SHH, ATRT), VUMC-ATRT-04 (MYC, ATRT), VUMC-EPN-06 (PFA, EPN), VUMC-EPN-05 (PFA, EPN), and VUMC-EPN-01 (PFA, EPN) were established from autopsy or resection material at the Amsterdam UMC (Vrije University Medical Center of Amsterdam, Amsterdam, the Netherlands).[38,56,87,88] Previously characterized and known mutations of the samples are shown in detail according to Table S4 also found in (shinyapps.io).[41,89] All patient material was collected according to national and institutional guidelines and in accordance with the declaration of Helsinki.

HSJD-DIPG-07, JHH-DIPG-01, SU-DIPG-IV, SU-DIPG-XXI, SU-pcGBM2, CHLA-ATRT-266, VUMC-DIPG-10, VUMC-DIPG-G, VUMC-ATRT-03, VUMC-ATRT-01, KNS-42, OPBG-GBM-001, VUMC-DIPG-11, and VUMC-DIPG-F were cultured at 37°C and 5% CO_2 in Tumor Stem Medium (TSM) consisting of 48% Neurobasal-A medium (Thermo Fisher, #10888022, Amsterdam, the Netherlands), 48% Dulbecco's modified Eagle's medium (DMEM)/F12 with Phenol Red without glutamine (Thermo Fisher, #31330095, Amsterdam, the Netherlands), 1% HEPES 1M (Thermo Fisher, #15630-080, Amsterdam, the Netherlands), 1% MEM Non-essential amino acid solution (Thermo Fisher, #11140050, Amsterdam, the Netherlands), 1% Sodium pyruvate 100 mM (Thermo Fisher, #11360039, Amsterdam, the Netherlands), 1% Glutamax (Thermo Fisher, #35050038, Amsterdam, the Netherlands) (TSM base). TSM base was supplemented with 2% B27 without vitamin A (Thermo Fisher, #12587010, Amsterdam, the Netherlands), 20 ng/mL human Epidermal Growth Factor (Peprotech, #AF-100-18B-1MG, Amsterdam, the Netherlands), 20 ng/mL human Basic Fibroblast Growth Factor (Peprotech, #AF-100-18B-1MG, Amsterdam, the Netherlands), 10 ng/mL human Platelet-derived Growth Factor AA (Peprotech, #100-13A-250μG, Amsterdam, the Netherlands), 10 ng/mL human Platelet-derived Growth Factor BB (Peprotech, #100-14B-250μG, Amsterdam, the Netherlands), 5,000 U/mL Heparin and 1% penicillin/streptomycin (Sigma Aldrich, P0781-100ML, Amsterdam, the Netherlands) (Complete TSM).

VUMC-ATRT-04, VUMC-EPN-06, VUMC-EPN-05, and VUMC-EPN-01 were cultured at 37°C and 5% CO_2 in TSM base supplemented with 10% heat-inactivated FBS (Sigma Aldrich, F0804, Amsterdam, the Netherlands) and 1% penicillin/streptomycin.

Cells were only used when confirmed mycoplasma negative with MycoAlert Mycoplasma Detection kit (Lonza, LT07-318) and

short-tandem repeat analysis with GenePrint 10 system (Promega, B9510, Leiden, the Netherlands) was performed to ensure cell line identities.

Viruses

Delta24-RGD, rQNestin34.5v1, R124, and rNDV-F0-GFP were produced as previously described.[25,30,34,90] Delta24-RGD viral stocks were titrated on HEK 293 cells using the Adeno-X Rapid Titer Kit (Takara,#632250), 12-well plates of HEK 293 cells were incubated in DMEM and 10% FBS, while 10-fold serial dilutions of the AdV were prepared and incubated with the cells for 48 h, the medium was then removed and cells were left to dry in hood for 5 min. The HEK 293 cells were then fixed with 100% methanol and incubated at −20°C for 10 min and rinsed three times. Mouse anti-hexon antibody (Takara,#632250) 1:1,000 in phosphate-buffered saline (PBS) was incubated with the cells for 1 h at 37°C and then rinsed and incubated with Rat anti-mouse (HRP-conjugated) (Takara,#632250) 1:500 for 1 h at 37°C and rinsed. DAP working solution was added to each well for 10 min at RT and washed. The infectious units (iu/mL) were calculated with optically counting the brown/black positive cells with the following formula: (infected cell/field) × (field/well))/(-volume virus (mL) × (dilution factor). The physical titer of Delta24-RGD was obtained through OD_{260} by lysing the viral stock in a 20-fold dilution in lysis solution (0.1% SDS, 10 mM Tris-Cl [pH 7.4], 1 mM EDTA), incubating at 56°C for 10 min, after which the disrupted virus solution was placed in a cuvette and the OD_{260} was determined. The concentration of the AdV was calculated in vp/mL by OD260 reading × dilution factor × 1.1 × 10^{12} = vp/mL.

rQNestin34.5v1 was titrated by plaque assay on Vero cells. Briefly, six-well plates of Vero cells were incubated for a day at 37°C at 5% CO_2 in DMEM and 10% FBS. The following day, 10-fold dilutions of the HSV stock were prepared (10^{-2} to 10^{-10}) and left for 60 to 90 min in the incubator with frequent rocking of the plates. After 90 min, the virus inoculum was removed and the wells were overlaid with 1% methylcellulose and incubated for 3–5 days until defined plaques were formed. The methylcellulose was then removed and the wells were stained with 1% crystal violet for 5 min at RT prior to rinsing and air drying. The number of plaque-forming units (PFU)/mL was determined by (the average number of plaques of each dilution multiplied by a factor of 10). The physical titer of the virus was measured as genome copies (G.C./mL) by quantifying the average G.C. of UL5 (F: 5′-GTG ATG CGA CTG GCG TTG G-3′, R: 5′-CAG TTT GTG GAC CGC TTT GT-3′) and glycoprotein D (US6) (F: 5′-GCG TGT TTA CCA CAT TCA GCC-3′, R: 5′-TCC GTC CAG TCG TTT ATC TTC A-3′) genes using standard curves for UL5 or US6 originating from 10-fold serial dilutions of plasmid containing UL5/US6 target sequences quantified by ABI-Plus 7000 Sequence Detector (ABI, USA) and analyzed using Sequence Detector V1.6 (PE Applied Biosystems, USA).

For R124, RV stocks were serially diluted in DMEM and 2% FBS and infected near-confluent HER 911 cells in six-well plates for 2-h incubation at 37°C, the medium was replaced with minimal essential medium (MEM), 12.5 mM $MgCl_2$, 2 mM GlutaMAX, and 0.5% agarose and plaques were counted six days post infection and PFU/mL was determined by (average number of dilutions × 10). The physical titer of R124 was measured through OD_{260} by lysing the viral stock as was described for Delta24-RGD. Vp/mL was estimated by empirical determined relation: 5.42 OD_{260} = 1mg of RV = 1.13 × 10^{13} RV particles.[91]

For rNDV-F0-GFP, viral stocks were titrated by endpoint dilution assay in Vero cells previously incubated overnight in DMEM and 2% FBS in 48-well plates. Ten-fold serial dilutions of the virus were prepared and used to infect the Vero cells for 2 h at 37°C, followed by addition of medium and monitoring cytopathic effects (CPE) for 1 to 4 weeks. Titers were calculated as tissue culture infectious dose (TCID) required to infect 50% of the cell monolayers by visualizing CPE and using the method of Reed and Muench.[92]

Cell viability assay

Single-cell suspensions were collected after cells were treated with accutase (Merck, A6964-100ML, Amsterdam, the Netherlands) and washed with PBS. Then, 2,000 cells/well were seeded in triplicate onto 96-well flat-bottom plates in 100 μL of the respective cell line's fresh culture media and left to incubate at 37°C and 5% CO_2 for 24 h. The cells were then infected with increasing doses of virus and left to incubate at 37°C and 5% CO_2 for 5 days. The concentration of the OVs is defined as MOI, which constitutes the number of infectious viral particles per cell. MOI ranges were for Delta24-RGD (MOI 50, 25, 12.5, 6.25, 3.125), for rQNestin34.5v1 (MOI 30, 10, 3.3, 1.1, 0.37, 0.12, 0.04), for R124 (MOI 300, 100, 33, 11, 3.7, 1.23, 0.41), and for rNDV-F0-GFP (MOI 10, 3.3, 1.1, 0.37, 0.12, 0.04, 0.01). During infections with rQNestin35.4v1, culture medium without heparin was applied in the assay to prevent non-cellular binding of HSV particles to soluble heparin, while in the cases in which FCS-supplemented culture medium was applied, the FCS concentration was reduced to 2% on the day of infection to prevent FCS interference with viral attachment. On day 5, infection was stopped, and cells were incubated for 10 min with CellTiter-Glo 2.0 assay reagent (Promega, #G924A, Leiden, the Netherlands), then the mixes were transferred into new clear flat-bottom black 96-well plates (Greiner, #655076, Mannheim, Germany) and luminescence was measured using a Tecan infinite M200 plate reader (Tecan) using the icontrol 1.10 software (Tecan). Background luminescence in the corresponding medium without cells was subtracted from experimental values. Values were normalized for calculating percentage of viable cells taking the values of uninfected cells as 100%. The mean values of the normalized data from two to four independent experiments per virus per cell line were used in a dose-normalized response with a variable slope and from the non-linear fit the effective concentrations needed to kill at least 50% of the cells (EC_{50}) for each virus was calculated using GraphPad Prism 9.

Immunofluorescence imaging

Cells infected with rQNestin34.5v1 and rNDV-F0-GFP in a 96-well plate were imaged after 5 days of infection with MOI 3.3 and 1.1,

respectively, using EVOS M5000 Imaging System (Invitrogen, USA) at ×100 magnification.

RNA-sequencing and analysis

Total RNA of non-infected cell lines was extracted and collected before the viability assay, using the miRvana miRNA isolation kit (Ambion, #AM1560, Landsmeer, the Netherlands) according to the manufacturers protocol. RNA quality and purity were assessed by the Agilent 2100 Bioanalyzer system using the Agilent RNA6000 Nano kit (Agilent, #5067-1511, CA, USA) and samples with RIN value of <7 were excluded from sequencing. The sample preparation was performed according to the protocol "NEBNext Ultra Directional RNA Library Prep Kit for Illumina" (NEB #E7420 S/L), briefly using oligo-dT magnetic beads mRNA was isolated from total RNA, followed by fragmentation and cDNA synthesis. After ligation of the sequencing adapters and PCR amplification, the quality and yield were measured with Fragment Analyzer. Clustering and DNA sequencing were performed with NovaSeq6000 following the manufacturer's protocol. Sequence reads were trimmed using cutadapt v2.10 removing adapter sequences. For each sample, the trimmed reads were mapped to the human GRCh38.p13 reference genome based on Burrow-Wheeler Transform using the default settings of STARv2.5.4. The BAM files were sorted and indexed with the samtools v1.10 package. All sequencing, adapter removal, trimming, and mapping and feature counting were performed by GenomeScan (Leiden, the Netherlands). The count matrix was loading in EdgeR package the gene counts with the same gene symbol were summarized using the R function "aggregate."[93] Then using the R function "filterByExpr," genes with less than 10 counts and not expressed in 70% of the samples or a total count of less than 15 counts across all samples were filtered out. The filtered genes were normalized through a trimmed mean of M-values (TMM) and counts per million (CPM) were computed followed by a log2 (CPM) transformation to be used in downstream analysis.

Viral sensitivity correlation

The correlation of the transcription level of genes and EC_{50} values was calculated by Spearman correlation per OV based on normalized gene expression of the tested cell lines. The correlation was executed using the "cor.test" function from the basic R package for a univariate Spearman correlation and genes with a p value <0.05 and ρ coefficient >0.5 were defined as significant genes correlating with resistance whereas genes with ρ coefficient <−0.5 were defined as genes correlating with sensitivity.

GO enrichment analysis

GO enrichment analysis was performed separately for the resistance correlated genes and sensitivity-related genes using the R package "cluster profiler."[94] The significant genes correlated with the sensitivity and resistance identified from the correlation analysis were used to query the GO database,[95] respectively. GO terms with a p-adjusted value (FDR method) < 0.05 were considered significantly enriched by the correlating genes in the corresponding groups.

Statistical analysis

For the *in vitro* experiments, the data were visualized as the mean ± standard deviation (SD), and the comparisons of mean EC_{50} values between the tumor entity groups were performed using a non-parametric Kruskal-Wallis test with Dunn's multiple comparison correction. All statistics for EC_{50} calculation and comparisons were performed using GraphPad Prism 9, while p values, ρ coefficients for the gene expression, correlation, and GO enrichment were performed using R.

DATA AND CODE AVAILABILITY

The expression data supporting the findings of this study are available in the GEO database with ascension number GEO: GSE260516.

SUPPLEMENTAL INFORMATION

Supplemental information can be found online at https://doi.org/10.1016/j.omton.2024.200804.

ACKNOWLEDGMENTS

The authors gratefully acknowledge the patients and their families who kindly donated tumor tissue. This project was funded by Inspire2live and Choir4hope to J.v.d.L., Stichting Kinderen Kankervrij (Kika) to E.H., and the Foundation Support Casper to M.L.M.L.

AUTHOR CONTRIBUTIONS

Conception and design: K.V., E.S., E.H., F.G.C., J.v.d.L., and M.L.M.L. Development of methodology: K.V., E.S., J.J., A.S, Y.L., L.V, P.W., D.S.M., M.H.M., B.v.d.H., R.C.H., and M.M.A. Acquisition of data: K.V. and P.W. Analysis and interpretation of data: K.V., E.S., J.J., E.H., F.G.C., J.v.d.L., and M.L.M.L. The first draft was written by K.V. Writing, review, and/or revision of the manuscript: K.V., E.S., E.H., F.G.C., J.v.d.L., M.L.M.L., M.H.M., V.K., B.v.d.H., R.C.H., E.A.C., and W.F.G. Study supervision: E.H., F.G.J.C., J.v.d.L., and M.L.M.L. All authors have read and approved the final version.

DECLARATION OF INTERESTS

The authors declare no competing interests.

REFERENCES

1. Ostrom, Q.T., Gittleman, H., Xu, J., Kromer, C., Wolinsky, Y., Kruchko, C., and Barnholtz-Sloan, J.S. (2016). CBTRUS statistical report: Primary brain and other central nervous system tumors diagnosed in the United States in 2009-2013. Neuro Oncol. *18*, v1–v75.
2. Braunstein, S., Raleigh, D., Bindra, R., Mueller, S., and Haas-Kogan, D. (2017). Pediatric high-grade glioma: current molecular landscape and therapeutic approaches. J. Neuro Oncol. *134*, 541–549.
3. Hargrave, D., Bartels, U., and Bouffet, E. (2006). Diffuse brainstem glioma in children: critical review of clinical trials. Lancet Oncol. *7*, 241–248.
4. Biegel, J.A. (2006). Molecular genetics of atypical teratoid/rhabdoid tumor. Neurosurg. Focus *20*, 1–7.
5. Fischer-Valuck, B.W., Chen, I., Srivastava, A.J., Floberg, J.M., Rao, Y.J., King, A.A., Shinohara, E.T., and Perkins, S.M. (2017). Assessment of the treatment approach and survival outcomes in a modern cohort of patients with atypical teratoid rhabdoid tumors using the National Cancer Database. Cancer *123*, 682–687.
6. Allen, J.C., Siffert, J., and Hukin, J. (1998). Clinical Manifestations of Childhood Ependymoma:A Multitude of Syndromes. Pediatr. Neurosurg. *28*, 49–55.

7. Pajtler, K.W., Witt, H., Sill, M., Jones, D.T.W., Hovestadt, V., Kratochwil, F., Wani, K., Tatevossian, R., Punchihewa, C., Johann, P., et al. (2015). Molecular Classification of Ependymal Tumors across All CNS Compartments, Histopathological Grades, and Age Groups. Cancer Cell *27*, 728–743.

8. Johann, P.D., Erkek, S., Zapatka, M., Kerl, K., Buchhalter, I., Hovestadt, V., Jones, D.T.W., Sturm, D., Hermann, C., Segura Wang, M., et al. (2016). Atypical Teratoid/Rhabdoid Tumors Are Comprised of Three Epigenetic Subgroups with Distinct Enhancer Landscapes. Cancer Cell *29*, 379–393.

9. Mackay, A., Burford, A., Carvalho, D., Izquierdo, E., Fazal-Salom, J., Taylor, K.R., Bjerke, L., Clarke, M., Vinci, M., Nandhabalan, M., et al. (2017). Integrated Molecular Meta-Analysis of 1,000 Pediatric High-Grade and Diffuse Intrinsic Pontine Glioma. Cancer Cell *32*, 520–537.e5.

10. Capper, D., Jones, D.T.W., Sill, M., Hovestadt, V., Schrimpf, D., Sturm, D., Koelsche, C., Sahm, F., Chavez, L., Reuss, D.E., et al. (2018). DNA methylation-based classification of central nervous system tumours. Nature *555*, 469–474.

11. Yeh, J.M., Ward, Z.J., Chaudhry, A., Liu, Q., Yasui, Y., Armstrong, G.T., Gibson, T.M., Howell, R., Hudson, M.M., Krull, K.R., et al. (2020). Life Expectancy of Adult Survivors of Childhood Cancer Over 3 Decades. JAMA Oncol. *6*, 350–357.

12. Uchida, H., Marzulli, M., Nakano, K., Goins, W.F., Chan, J., Hong, C.-S., Mazzacurati, L., Yoo, J.Y., Haseley, A., Nakashima, H., et al. (2013). Effective Treatment of an Orthotopic Xenograft Model of Human Glioblastoma Using an EGFR-retargeted Oncolytic Herpes Simplex Virus. Mol. Ther. *21*, 561–569.

13. Todo, T., Feigenbaum, F., Rabkin, S.D., Lakeman, F., Newsome, J.T., Johnson, P.A., Mitchell, E., Belliveau, D., Ostrove, J.M., and Martuza, R.L. (2000). Viral Shedding and Biodistribution of G207, a MuItimutated, Conditionally Replicating Herpes Simplex Virus Type 1, after Intracerebral Inoculation in Aotus. Mol. Ther. *2*, 588–595.

14. Markert, J.M., Medlock, M.D., Rabkin, S.D., Gillespie, G.Y., Todo, T., Hunter, W.D., Palmer, C.A., Feigenbaum, F., Tornatore, C., Tufaro, F., and Martuza, R.L. (2000). Conditionally replicating herpes simplex virus mutant, G207 for the treatment of malignant glioma: results of a phase I trial. Gene Ther. *7*, 867–874.

15. Zamarin, D., Holmgaard, R.B., Subudhi, S.K., Park, J.S., Mansour, M., Palese, P., Merghoub, T., Wolchok, J.D., and Allison, J.P. (2014). Localized oncolytic virotherapy overcomes systemic tumor resistance to immune checkpoint blockade immunotherapy. Sci. Transl. Med. *6*, 226ra32.

16. Nassiri, F., Patil, V., Yefet, L.S., Singh, O., Liu, J., Dang, R.M.A., Yamaguchi, T.N., Daras, M., Cloughesy, T.F., Colman, H., et al. (2023). Oncolytic DNX-2401 virotherapy plus pembrolizumab in recurrent glioblastoma: a phase 1/2 trial. Nat. Med. *29*, 1370–1378.

17. Ling, A.L., Solomon, I.H., Landivar, A.M., Nakashima, H., Woods, J.K., Santos, A., Masud, N., Fell, G., Mo, X., Yilmaz, A.S., et al. (2023). Clinical trial links oncolytic immunoactivation to survival in glioblastoma. Nature *623*, 157–166.

18. Gállego Pérez-Larraya, J., Garcia-Moure, M., Labiano, S., Patiño-García, A., Dobbs, J., Gonzalez-Huarriz, M., Zalacain, M., Marrodan, L., Martinez-Velez, N., Puigdelloses, M., et al. (2022). Oncolytic DNX-2401 Virus for Pediatric Diffuse Intrinsic Pontine Glioma. N. Engl. J. Med. *386*, 2471–2481.

19. Yang, W.Q., Senger, D., Muzik, H., Shi, Z.Q., Johnson, D., Brasher, P.M.A., Rewcastle, N.B., Hamilton, M., Rutka, J., Wolff, J., et al. (2003). Reovirus prolongs survival and reduces the frequency of spinal and leptomeningeal metastases from medulloblastoma. Cancer Res. *63*, 3162–3172.

20. Waters, A.M., Johnston, J.M., Reddy, A.T., Fiveash, J., Madan-Swain, A., Kachurak, K., Bag, A.K., Gillespie, G.Y., Markert, J.M., and Friedman, G.K. (2017). Rationale and Design of a Phase 1 Clinical Trial to Evaluate HSV G207 Alone or with a Single Radiation Dose in Children with Progressive or Recurrent Malignant Supratentorial Brain Tumors. Hum. Gene Ther. Clin. Dev. *28*, 7–16.

21. Csatary, L.K., Gosztonyi, G., Szeberenyi, J., Fabian, Z., Liszka, V., Bodey, B., and Csatary, C.M. (2004). MTH-68/H oncolytic viral treatment in human high-grade gliomas. J. Neuro Oncol. *67*, 83–93.

22. Suzuki, K., Fueyo, J., Krasnykh, V., Reynolds, P.N., Curiel, D.T., and Alemany, R. (2001). A conditionally replicative adenovirus with enhanced infectivity shows improved oncolytic potency. Clin. Cancer Res. *7*, 120–126.

23. Martuza, R.L., Malick, A., Markert, J.M., Ruffner, K.L., and Coen, D.M. (1991). Experimental therapy of human glioma by means of a genetically engineered virus mutant. Science *252*, 854–856.

24. Krupkova, O., Jr., Loja, T., Zambo, I., and Veselska, R. (2010). Nestin expression in human tumors and tumor cell lines. Neoplasma *57*, 291–298.

25. Kambara, H., Okano, H., Chiocca, E.A., and Saeki, Y. (2005). An oncolytic HSV-1 mutant expressing ICP34.5 under control of a nestin promoter increases survival of animals even when symptomatic from a brain tumor. Cancer Res. *65*, 2832–2839.

26. Norman, K.L., Hirasawa, K., Yang, A.-D., Shields, M.A., and Lee, P.W.K. (2004). Reovirus oncolysis: the Ras/RalGEF/p38 pathway dictates host cell permissiveness to reovirus infection. Proc. Natl. Acad. Sci. USA *101*, 11099–11104.

27. Shmulevitz, M., Marcato, P., and Lee, P.W.K. (2010). Activated Ras signaling significantly enhances reovirus replication and spread. Cancer Gene Ther. *17*, 69–70.

28. Marcato, P., Shmulevitz, M., Pan, D., Stoltz, D., and Lee, P.W. (2007). Ras Transformation Mediates Reovirus Oncolysis by Enhancing Virus Uncoating, Particle Infectivity, and Apoptosis-dependent Release. Mol. Ther. *15*, 1522–1530.

29. Park, E.-H., Park, E.H., Cho, I.-R., Srisuttee, R., Min, H.-J., Oh, M.-J., Jeong, Y.-J., Jhun, B.H., Johnston, R.N., Lee, S., et al. (2010). CUG2, a novel oncogene confers reoviral replication through Ras and p38 signaling pathway. Cancer Gene Ther. *17*, 307–314.

30. van den Hengel, S.K., Balvers, R.K., Dautzenberg, I.J.C., van den Wollenberg, D.J.M., Kloezeman, J.J., Lamfers, M.L., Sillivis-Smit, P.A.E., and Hoeben, R.C. (2013). Heterogeneous reovirus susceptibility in human glioblastoma stem-like cell cultures. Cancer Gene Ther. *20*, 507–513.

31. Krishnamurthy, S., Takimoto, T., Scroggs, R.A., and Portner, A. (2006). Differentially regulated interferon response determines the outcome of Newcastle disease virus infection in normal and tumor cell lines. J. Virol. *80*, 5145–5155.

32. García-Romero, N., Palacín-Aliana, I., Esteban-Rubio, S., Madurga, R., Rius-Rocabert, S., Carrión-Navarro, J., Presa, J., Cuadrado-Castano, S., Sánchez-Gómez, P., García-Sastre, A., et al. (2020). Newcastle Disease Virus (NDV) Oncolytic Activity in Human Glioma Tumors Is Dependent on CDKN2A-Type I IFN Gene Cluster Codeletion. Cells *9*, 1405.

33. Buijs, P., van Nieuwkoop, S., Vaes, V., Fouchier, R., van Eijck, C., and van den Hoogen, B. (2015). Recombinant Immunomodulating Lentogenic or Mesogenic Oncolytic Newcastle Disease Virus for Treatment of Pancreatic Adenocarcinoma. Viruses *7*, 2980–2998.

34. Buijs, P.R.A., van Eijck, C.H.J., Hofland, L.J., Fouchier, R.A.M., and van den Hoogen, B.G. (2014). Different responses of human pancreatic adenocarcinoma cell lines to oncolytic Newcastle disease virus infection. Cancer Gene Ther. *21*, 24–30.

35. Lee, J., Kotliarova, S., Kotliarov, Y., Li, A., Su, Q., Donin, N.M., Pastorino, S., Purow, B.W., Christopher, N., Zhang, W., et al. (2006). Tumor stem cells derived from glioblastomas cultured in bFGF and EGF more closely mirror the phenotype and genotype of primary tumors than do serum-cultured cell lines. Cancer Cell *9*, 391–403.

36. Wakimoto, H., Mohapatra, G., Kanai, R., Curry, W.T., Jr., Yip, S., Nitta, M., Patel, A.P., Barnard, Z.R., Stemmer-Rachamimov, A.O., Louis, D.N., et al. (2012). Maintenance of primary tumor phenotype and genotype in glioblastoma stem cells. Neuro Oncol. *14*, 132–144.

37. Pinto, B., Henriques, A.C., Silva, P.M.A., and Bousbaa, H. (2020). Three-dimensional spheroids as in vitro preclinical models for cancer research. Pharmaceutics *12*, 1186. https://doi.org/10.3390/pharmaceutics12121186.

38. Meel, M.H., Sewing, A.C.P., Waranecki, P., Metselaar, D.S., Wedekind, L.E., Koster, J., van Vuurden, D.G., Kaspers, G.J.L., and Hulleman, E. (2017). Culture methods of diffuse intrinsic pontine glioma cells determine response to targeted therapies. Exp. Cell Res. *360*, 397–403.

39. Paassen, I., Williams, J., Ríos Arceo, C., Ringnalda, F., Mercer, K.S., Buhl, J.L., Moreno, N., Federico, A., Franke, N.E., Kranendonk, M., et al. (2023). Atypical teratoid/rhabdoid tumoroids reveal subgroup-specific drug vulnerabilities. Oncogene *42*, 1661–1671.

40. Peterziel, H., Jamaladdin, N., ElHarouni, D., Gerloff, X.F., Herter, S., Fiesel, P., Berker, Y., Blattner-Johnson, M., Schramm, K., Jones, B.C., et al. (2022). Drug sensitivity profiling of 3D tumor tissue cultures in the pediatric precision oncology program INFORM. npj Precis. Oncol. *6*, 94.

41. Sun, C.X., Daniel, P., Bradshaw, G., Shi, H., Loi, M., Chew, N., Parackal, S., Tsui, V., Liang, Y., Koptyra, M., et al. (2023). Generation and multi-dimensional profiling of a childhood cancer cell line atlas defines new therapeutic opportunities. Cancer Cell *41*, 660–677.e7.

42. Martínez-Vélez, N., Garcia-Moure, M., Marigil, M., González-Huarriz, M., Puigdelloses, M., Gallego Pérez-Larraya, J., Zalacaín, M., Marrodán, L., Varela-Guruceaga, M., Laspidea, V., et al. (2019). The oncolytic virus Delta-24-RGD elicits an antitumor effect in pediatric glioma and DIPG mouse models. Nat. Commun. *10*, 2235.

43. Fusco, D., Forghieri, C., and Campadelli-Fiume, G. (2005). The pro-fusion domain of herpes simplex virus glycoprotein D (gD) interacts with the gD N terminus and is displaced by soluble forms of viral receptors. Proc. Natl. Acad. Sci. USA *102*, 9323–9328.

44. Bacsa, S., Karasneh, G., Dosa, S., Liu, J., Valyi-Nagy, T., and Shukla, D. (2011). Syndecan-1 and syndecan-2 play key roles in herpes simplex virus type-1 infection. J. Gen. Virol. *92*, 733–743.

45. Satoh, T., Arii, J., Suenaga, T., Wang, J., Kogure, A., Uehori, J., Arase, N., Shiratori, I., Tanaka, S., Kawaguchi, Y., et al. (2008). PILRalpha is a herpes simplex virus-1 entry coreceptor that associates with glycoprotein B. Cell *132*, 935–944.

46. Arii, J., Goto, H., Suenaga, T., Oyama, M., Kozuka-Hata, H., Imai, T., Minowa, A., Akashi, H., Arase, H., Kawaoka, Y., and Kawaguchi, Y. (2010). Non-muscle myosin IIA is a functional entry receptor for herpes simplex virus-1. Nature *467*, 859–862.

47. O'Donnell, S.M., Hansberger, M.W., and Dermody, T.S. (2003). Viral and cellular determinants of apoptosis induced by mammalian reovirus. Int. Rev. Immunol. *22*, 477–503.

48. Konopka-Anstadt, J.L., Mainou, B.A., Sutherland, D.M., Sekine, Y., Strittmatter, S.M., and Dermody, T.S (2014). The Nogo Receptor NgR1 Mediates Infection by Mammalian Reovirus. Cell Host Microbe *15*, 681–691.

49. Ran, Z., Qiankai, S., Zongxi, H., Zhen, F., Hui, A., Linna, C., Le, L., Tianyi, L., Junfeng, S., and Shengwang, L. (2021). Newcastle Disease Virus Entry into Chicken Macrophages via a pH-Dependent, Dynamin and Caveola-Mediated Endocytic Pathway That Requires Rab5. J. Virol. *95*, e0228820.

50. Cantín, C., Holguera, J., Ferreira, L., Villar, E., and Muñoz-Barroso, I. (2007). Newcastle disease virus may enter cells by caveolae-mediated endocytosis. J. Gen. Virol. *88*, 559–569.

51. Ghajar-Rahimi, G., Kang, K.-D., Totsch, S.K., Gary, S., Rocco, A., Blitz, S., Kachurak, K., Chambers, M.R., Li, R., Beierle, E., et al. (2022). Clinical Advances in Oncolytic Virotherapy for Pediatric Brain Tumors. Pharmacology & therapeutics *239*, 108193.

52. de la Nava, D., Selvi, K.M., and Alonso, M.M. (2022). Immunovirotherapy for Pediatric Solid Tumors: A Promising Treatment That is Becoming a Reality. Front. Immunol. *13*, 866892.

53. Garcia-Moure, M., Gonzalez-Huarriz, M., Labiano, S., Guruceaga, E., Bandres, E., Zalacain, M., Marrodan, L., de Andrea, C., Villalba, M., Martinez-Velez, N., et al. (2021). Delta-24-RGD, an Oncolytic Adenovirus, Increases Survival and Promotes Proinflammatory Immune Landscape Remodeling in Models of AT/RT and CNS-PNET. Clin. Cancer Res. *27*, 1807–1820.

54. Friedman, G.K., Moore, B.P., Nan, L., Kelly, V.M., Etminan, T., Langford, C.P., Xu, H., Han, X., Markert, J.M., Beierle, E.A., and Gillespie, G.Y. (2016). Pediatric medulloblastoma xenografts including molecular subgroup 3 and CD133+ and CD15+ cells are sensitive to killing by oncolytic herpes simplex viruses. Neuro Oncol. *18*, 227–235.

55. Varela-Guruceaga, M., Tejada-Solís, S., García-Moure, M., Fueyo, J., Gomez-Manzano, C., Patiño-García, A., and Alonso, M.M. (2018). Oncolytic Viruses as Therapeutic Tools for Pediatric Brain Tumors. Cancers *10*, 226.

56. Meel, M.H., de Gooijer, M.C., Guillén Navarro, M., Waranecki, P., Breur, M., Buil, L.C.M., Wedekind, L.E., Twisk, J.W.R., Koster, J., Hashizume, R., et al. (2018). MELK Inhibition in Diffuse Intrinsic Pontine Glioma. Clin. Cancer Res. *24*, 5645–5657.

57. Ntafoulis, I., Kleijn, A., Ju, J., Jimenez-Cowell, K., Fabro, F., Klein, M., Chi Yen, R.T., Balvers, R.K., Li, Y., Stubbs, A.P., et al. (2023). Ex vivo drug sensitivity screening predicts response to temozolomide in glioblastoma patients and identifies candidate biomarkers. Br. J. Cancer *129*, 1327–1338. https://doi.org/10.1038/s41416-023-02402-y.

58. Bhatt, D.K., Chammas, R., and Daemen, T. (2021). Resistance mechanisms influencing oncolytic virotherapy, a systematic analysis. Vaccines *9*, 1166. https://doi.org/10.3390/vaccines9101166.

59. Karasneh, G.A., Ali, M., and Shukla, D. (2011). An Important Role for Syndecan-1 in Herpes Simplex Virus Type-1 Induced Cell-to-Cell Fusion and Virus Spread. PLoS One *6*, e25252.

60. Jennings, M.R., and Parks, R.J. (2023). Human Adenovirus Gene Expression and Replication Is Regulated through Dynamic Changes in Nucleoprotein Structure throughout Infection. Viruses *15*, 161. https://doi.org/10.3390/v15010161.

61. Miller, D.L., Myers, C.L., Rickards, B., Coller, H.A., and Flint, S.J. (2007). Adenovirus type 5 exerts genome-wide control over cellular programs governing proliferation, quiescence, and survival. Genome Biol. *8*, R58.

62. Komatsu, T., Haruki, H., and Nagata, K. (2011). Cellular and viral chromatin proteins are positive factors in the regulation of adenovirus gene expression. Nucleic Acids Res. *39*, 889–901.

63. Dybas, J.M., Lum, K.K., Kulej, K., Reyes, E.D., Lauman, R., Charman, M., Purman, C.E., Steinbock, R.T., Grams, N., Price, A.M., et al. (2021). Adenovirus Remodeling of the Host Proteome and Host Factors Associated with Viral Genomes. mSystems *6*, 10–1128.

64. Donovan-Banfield, I., Turnell, A.S., Hiscox, J.A., Leppard, K.N., and Matthews, D.A. (2020). Deep splicing plasticity of the human adenovirus type 5 transcriptome drives virus evolution. Commun. Biol. *3*, 124.

65. Palmer, D.H., Chen, M.-J., Searle, P.F., Kerr, D.J., and Young, L.S. (2005). Inhibition of NF-κB enhances the cytotoxicity of virus-directed enzyme prodrug therapy and oncolytic adenovirus cancer gene therapy. Gene Ther. *12*, 1187–1197.

66. Shen, B.H., and Hermiston, T.W. (2005). Effect of hypoxia on Ad5 infection, transgene expression and replication. Gene Ther. *12*, 902–910. https://doi.org/10.1038/sj.gt.3302448.

67. Liikanen, I., Monsurrò, V., Ahtiainen, L., Raki, M., Hakkarainen, T., Diaconu, I., Escutenaire, S., Hemminki, O., Dias, J.D., Cerullo, V., et al. (2011). Induction of Interferon Pathways Mediates In Vivo Resistance to Oncolytic Adenovirus. Mol. Ther. *19*, 1858–1866. https://doi.org/10.1038/mt.2011.144.

68. Carmichael, J.C., Yokota, H., Craven, R.C., Schmitt, A., and Wills, J.W. (2018). The HSV-1 mechanisms of cell-to-cell spread and fusion are critically dependent on host PTP1B. PLoS Pathog. *14*, e1007054.

69. Weed, D.J., and Nicola, A.V. (2017). Herpes simplex virus Membrane Fusion. Adv. Anat. Embryol. Cell Biol. *223*, 29–47.

70. Zhou, X., Wang, L., Zou, W., Chen, X., Roizman, B., and Zhou, G.G. (2020). hnRNPA2B1 Associated with Recruitment of RNA into Exosomes Plays a Key Role in Herpes Simplex Virus 1 Release from Infected Cells. J. Virol. *94*, e00367-20.

71. Zhu, L., and Jones, C. (2018). The canonical Wnt/β-catenin signaling pathway stimulates herpes simplex virus 1 productive infection. Virus Res. *256*, 29–37.

72. Campbell, S.L., Khosravi-Far, R., Rossman, K.L., Clark, G.J., and Der, C.J. (1998). Increasing complexity of Ras signaling. Oncogene *17*, 1395–1413.

73. Scheffzek, K., Ahmadian, M.R., Kabsch, W., Wiesmüller, L., Lautwein, A., Schmitz, F., and Wittinghofer, A. (1997). The Ras-RasGAP complex: structural basis for GTPase activation and its loss in oncogenic Ras mutants. Science *277*, 333–338.

74. Kominsky, D.J., Bickel, R.J., and Tyler, K.L. (2002). Reovirus-Induced Apoptosis Requires Mitochondrial Release of Smac/DIABLO and Involves Reduction of Cellular Inhibitor of Apoptosis Protein Levels. J. Virol. *76*, 11414–11424.

75. Sánchez-Felipe, L., Villar, E., and Muñoz-Barroso, I. (2014). Entry of Newcastle Disease Virus into the host cell: Role of acidic pH and endocytosis. Biochim. Biophys. Acta *1838*, 300–309.

76. Tan, L., Zhang, Y., Zhan, Y., Yuan, Y., Sun, Y., Qiu, X., Meng, C., Song, C., Liao, Y., and Ding, C. (2016). Newcastle disease virus employs macropinocytosis and Rab5a-dependent intracellular trafficking to infect DF-1 cells. Oncotarget *7*, 86117–86133.

77. Samal, S.K. (2011). Newcastle disease and related avian paramyxoviruses. The biology of paramyxoviruses *1*, 69–114.

78. Li, Y., Jiang, W., Niu, Q., Sun, Y., Meng, C., Tan, L., Song, C., Qiu, X., Liao, Y., and Ding, C. (2019). eIF2α-CHOP-BCl-2/JNK and IRE1α-XBP1/JNK signaling promote apoptosis and inflammation and support the proliferation of Newcastle disease virus. Cell Death Dis. *10*, 891.

79. Cheng, J.-H., Sun, Y.-J., Zhang, F.-Q., Zhang, X.-R., Qiu, X.-S., Yu, L.-P., Wu, Y.-T., and Ding, C. (2016). Newcastle disease virus NP and P proteins induce autophagy via

the endoplasmic reticulum stress-related unfolded protein response. Sci. Rep. *6*, 24721.

80. Meng, C., Zhou, Z., Jiang, K., Yu, S., Jia, L., Wu, Y., Liu, Y., Meng, S., and Ding, C. (2012). Newcastle disease virus triggers autophagy in U251 glioma cells to enhance virus replication. Arch. Virol. *157*, 1011–1018. https://doi.org/10.1007/s00705-012-1270-6.

81. Stavrakaki, E., van den Bossche, W.B.L., Vogelezang, L.B., Teodosio, C., Mustafa, D.M., van Dongen, J.J.M., Dirven, C.M.F., Balvers, R.K., and Lamfers, M.L. (2024). An autologous ex vivo model for exploring patient-specific responses to viro-immunotherapy in glioblastoma. Cell Rep. Methods *4*, 100716. https://doi.org/10.1016/j.crmeth.2024.100716.

82. Jansen, M.H.A., Lagerweij, T., Sewing, A.C.P., Vugts, D.J., van Vuurden, D.G., Molthoff, C.F.M., Caretti, V., Veringa, S.J.E., Petersen, N., Carcaboso, A.M., et al. (2016). Bevacizumab Targeting Diffuse Intrinsic Pontine Glioma: Results of 89Zr-Bevacizumab PET Imaging in Brain Tumor Models. Mol. Cancer Therapeut. *15*, 2166–2174.

83. Taylor, I.C., Hütt-Cabezas, M., Brandt, W.D., Kambhampati, M., Nazarian, J., Chang, H.T., Warren, K.E., Eberhart, C.G., and Raabe, E.H. (2015). Disrupting NOTCH Slows Diffuse Intrinsic Pontine Glioma Growth, Enhances Radiation Sensitivity, and Shows Combinatorial Efficacy With Bromodomain Inhibition. J. Neuropathol. Exp. Neurol. *74*, 778–790.

84. Grasso, C.S., Tang, Y., Truffaux, N., Berlow, N.E., Liu, L., Debily, M.-A., Quist, M.J., Davis, L.E., Huang, E.C., Woo, P.J., et al. (2015). Functionally defined therapeutic targets in diffuse intrinsic pontine glioma. Nat. Med. *21*, 827.

85. Kang, M.H., Smith, M.A., Morton, C.L., Keshelava, N., Houghton, P.J., and Reynolds, C.P. (2011). National Cancer Institute pediatric preclinical testing program: model description for in vitro cytotoxicity testing. Pediatr. Blood Cancer *56*, 239–249.

86. Takeshita, I., Takaki, T., Kuramitsu, M., Nagasaka, S., Machi, T., Ogawa, H., Egami, H., Mannoji, H., Fukui, M., and Kitamura, K. (1987). Characteristics of an Established Human Glioma Cell Line, KNS-42. Neurol. Med.-Chir. *27*, 581–587.

87. Meel, M.H., Guillén Navarro, M., de Gooijer, M.C., Metselaar, D.S., Waranecki, P., Breur, M., Lagerweij, T., Wedekind, L.E., Koster, J., van de Wetering, M.D., et al. (2020). MEK/MELK inhibition and blood-brain barrier deficiencies in atypical teratoid/rhabdoid tumors. Neuro Oncol. *22*, 58–69.

88. Meel, M.H., Metselaar, D.S., Waranecki, P., Kaspers, G.J.L., and Hulleman, E. (2018). An efficient method for the transduction of primary pediatric glioma neurospheres. MethodsX *5*, 173–183.

89. Metselaar, D.S., du Chatinier, A., Meel, M.H., ter Huizen, G., Waranecki, P., Goulding, J.R., Bugiani, M., Koster, J., Kaspers, G.J.L., and Hulleman, E. (2022). AURKA and PLK1 inhibition selectively and synergistically block cell cycle progression in diffuse midline glioma. iScience *25*, 104398.

90. Lamfers, M.L.M., Grill, J., Dirven, C.M.F., Van Beusechem, V.W., Geoerger, B., Van Den Berg, J., Alemany, R., Fueyo, J., Curiel, D.T., Vassal, G., et al. (2002). Potential of the conditionally replicative adenovirus Ad5-Delta24RGD in the treatment of malignant gliomas and its enhanced effect with radiotherapy. Cancer Res. *62*, 5736–5742.

91. Smith, R.E., Zweerink, H.J., and Joklik, W.K. (1969). Polypeptide Components of Virions, Top Component and Cores of Reovirus Type 3. Virology *39*, 791–810.

92. REED, L.J., and Muench, H. (1938). A SIMPLE METHOD OF ESTIMATING FIFTY PER CENT ENDPOINTS. Am. J. Epidemiol. *27*, 493–497.

93. Robinson, M.D., McCarthy, D.J., and Smyth, G.K. (2010). edgeR: a Bioconductor package for differential expression analysis of digital gene expression data. Bioinformatics *26*, 139–140.

94. Yu, G., Wang, L.-G., Han, Y., and He, Q.-Y. (2012). clusterProfiler: an R package for comparing biological themes among gene clusters. OMICS *16*, 284–287.

95. Gene Ontology Consortium, Aleksander, S.A., Balhoff, J., Carbon, S., Cherry, J.M., Drabkin, H.J., Ebert, D., Feuermann, M., Gaudet, P., Harris, N.L., et al. (2023). The Gene Ontology knowledgebase in 2023. Genetics *224*, iyad031.

OMTON, Volume *32*

Supplemental information

The heterogeneous sensitivity of pediatric brain tumors to different oncolytic viruses is predicted by unique gene expression profiles

Konstantinos Vazaios, Eftychia Stavrakaki, Lisette B. Vogelezang, Jie Ju, Piotr Waranecki, Dennis S. Metselaar, Michaël H. Meel, Vera Kemp, Bernadette G. van den Hoogen, Rob C. Hoeben, E. Antonio Chiocca, William F. Goins, Andrew Stubbs, Yunlei Li, Marta M. Alonso, Friso G. Calkoen, Esther Hulleman, Jasper van der Lugt, and Martine L.M. Lamfers

Table S1. EC50 values

		**Delta24-RGD EC50s**[a]	
Cell-culture	Best-fit values EC50	95% CI (profile likelihood) EC50	R squared
HSJD-DIPG-07	4.426	2.450 to 6.473	0.8976
VUMC-DIPG-10	2.638	2.096 to 3.091	0.9655
VUMC-DIPG-G	51.15	34.92 to 82.13	0.86
SU-DIPG-XXI	31.97	25.07 to 40.99	0.9449
SU-DIPG-IV	22.78	19.31 to 27.16	0.9509
SU-pcGBM2	26.99	18.38 to 42.54	0.8031
JHH-DIPG-01	4.083	3.704 to 4.486	0.9898
VUMC-ATRT-03	9.47	5.923 to 14.02	0.9254
VUMC-ATRT-01	5.482	2.286 to 10.06	0.7782
CHLA-ATRT-266	4.841	3.428 to 6.457	0.9373
VUMC-ATRT-04	4.47	1.604 to 8.915	0.7757
VUMC-EPN-06	6.61	5.768 to 7.517	0.9805
VUMC-EPN-05	14.03	8.664 to 22.92	0.8827
VUMC-EPN-01	10.85	5.693 to 19.64	0.8732

a. Effective concentration needed to kill 50% of the cells

		**rQNestin 34.5v1 EC50s**[a]	
Cell-culture	Best-fit values EC50	95% CI (profile likelihood) EC50	R squared
HSJD-DIPG-07	0.7853	0.5549 to 1.044	0.9357
VUMC-DIPG-10	0.6459	0.5720 to 0.7291	0.9926
VUMC-DIPG-G	0.1786	0.1412 to 0.2262	0.9716
SU-DIPG-XXI	0.5347	0.4937 to 0.5793	0.9968
SU-DIPG-IV	0.07856	0.04815 to 0.1269	0.8893
SU-pcGBM2	0.07621	0.04999 to 0.1149	0.912
JHH-DIPG-01	0.8354	0.7122 to 0.9788	0.9851
VUMC-ATRT-03	out of bounds	???	???
VUMC-ATRT-01	1,121	0.7109 to 1.783	0.9276
CHLA-ATRT-266	8,412	6.128 to 11.72	0.9376
VUMC-ATRT-04	0.1326	0.08301 to 0.2102	0.9163
VUMC-EPN-06	0.3537	0.2498 to 0.5005	0.9541
VUMC-EPN-05	89.96	22.75 to 148240976	0.3554
VUMC-EPN-01	26.62	17.64 to 46.68	0.9278

a. Effective concentration needed to kill 50% of the cells

R124 EC50s[a]

Cell-culture	Best-fit values EC50	95% CI (profile likelihood) EC50	R squared
HSJD-DIPG-07	198.1	133.4 to 331.1	0.8738
VUMC-DIPG-10	157.1	121.4 to 212.2	0.9291
VUMC-DIPG-G	35.77	21.64 to 68.00	0.8925
SU-DIPG-XXI	471.6	??? to 2977	0.5478
SU-DIPG-IV	218.4	158.9 to 317.5	0.9495
SU-pcGBM2	~ 0.1357	(Very wide)	0.9748
JHH-DIPG-01	150.1	111.7 to 212.4	0.9237
VUMC-ATRT-03	0.3023	0.1123 to 0.7260	0.644
VUMC-ATRT-01	717.9	354.1 to 2414	0.8088
CHLA-ATRT-266	4,408	3.342 to 5.834	0.9693
VUMC-ATRT-04	8.26	5.027 to 13.92	0.9142
VUMC-EPN-06	283.5	189.6 to 575.1	0.5468
VUMC-EPN-05	461.1	309.2 to 865.3	0.9063
VUMC-EPN-01	197.3	111.5 to 482.2	0.7908

a. Effective concentration needed to kill 50% of the cells

rNDV-F0-GFP EC50s[a]

Cell-culture	Best-fit values EC50	95% CI (profile likelihood) EC50	R squared
HSJD-DIPG-07	5.819	4.473 to 7.788	0.9137
VUMC-DIPG-10	6.88	5.573 to 8.697	0.9314
VUMC-DIPG-G	4.596	3.102 to 7.560	0.9156
SU-DIPG-XXI	12.15	5.972 to 56.50	0.6026
SU-DIPG-IV	1.475	1.183 to 1.837	0.971
SU-pcGBM2	1.833	1.116 to 3.154	0.871
JHH-DIPG-01	4.379	2.660 to 8.542	0.8091
VUMC-ATRT-03	3.504	2.536 to 4.989	0.916
VUMC-ATRT-01	3.445	2.156 to 6.190	0.8192
CHLA-ATRT-266	75.95	20.31 to 1701	0.7118
VUMC-ATRT-04	2.532	1.520 to 4.443	0.833
VUMC-EPN-06	8.343	6.382 to 11.68	0.8625
VUMC-EPN-05	4.114	2.723 to 6.788	0.8613
VUMC-EPN-01	2.797	2.171 to 3.667	0.9588

a. Effective concentration needed to kill 50% of the cells

Table S2. Significantly correlating Genes

Table S3. Gene Ontology enrichment

Table S4. Known genetic mutations of cell models used in this study

Cell-culture	Entity (Driver mutation)	Other mutations
HSJD-DIPG-07	HGG (H3.3K27M)	CREBBP, MYC, PI3KCA, ACVR1, LRP1B, PPM1B, BRAF, CCND2
VUMC-DIPG-10	HGG (H3WT)	TP53, NF1, MYCN, KRAS, PPM1D
VUMC-DIPGG-G	HGG (H3.3K27M)	TP53, RB1, TERT, PTEN, MGMT, CDKN2A/B
SU-DIPG-XXI	HGG (H3.1K27M)	PDGFRA, ACVR1, MCL1
SU-DIPG-IV	HGG (H3.1K27M)	TP53, PIK3CA, LRP1B, MDM4, ACVR1
SU-pcGBM2	HGG (H3WT)	TP53, NF1, PIK3CA, TERT, RB1
JHH-DIPG-01	HGG (H3.3K27M)	TP53, MYC, MDM4, MET, CDK6, PI3KCA, PTEN
VUMC-ATRT-03	AT/RT-SHH (SMARCB1)	CDKN2A/B, EGFR, CDK6, BRAF, MDM4
VUMC-ATRT-01	AT/RT-SHH (SMARCB1)	MDM4, FGFR3
CHLA-ATRT-266	AT/RT-MYC (SMARCB1)	CREBBP, ARID1A, CDKN2A, FLT4, IGF2
VUMC-ATRT-04	AT/RT-MYC (SMARCB1)	
VUMC-EPN-06	EPN-PFA	
VUMC-EPN-05	EPN-PFA	
VUMC-EPN-01	EPN-PFA	
KNS-42	HGG (H3.3G34)	TP53, PI3KCA, TERT, MLC1
VUMC-DIPG-11	HGG (H3.3K27M)	TP53, MYB, RB1, PDGFRA, CCND1
VUMC-DIPG-F	HGG (H3.3K27M)	MYCN, MET, RB1, PPM1D, BRAF, MDM2
OPBG-GBM-001	HGG (H3.3G34)	TP53, PI3KCA, PDGFRA, BCOR, ATRX

Table S5. Infectious and physical titers of the OVs

OV	physical titer	infectious titer
Delta24-RGD	4.20x10^12 (vp/ml)	9.37 x10^10 (iu/ml)
rQNestin 34.5v1	7.9 x 10^{11} (GC/ml)	5.2 x 10^9 (PFU/ml)
R124	1.87x10^13 (vp/ml)	1.43 x10^11 (PFU/ml)
rNDV-F0-GFP	N/A	1.6 x10^8 (TCID50/ml)

Table S6. RIN values of RNA samples

RNA sample	RNA Integrity Number (RIN)	rRNA Ratio [28s/18s]
HSJD-DIPG-07	9.7	1.6
VUMC-DIPG-10	9.8	1.9
VUMC-DIPGG-G	9.6	1.6
SU-DIPG-XXI	9.5	1.7
SU-DIPG-IV	9.5	1.7
SU-pcGBM2	9.5	1.6
JHH-DIPG-01	9.9	1.7
VUMC-ATRT-03	9.4	1.7
VUMC-ATRT-01	9.5	1.8
CHLA-ATRT-266	9.5	1.8
VUMC-ATRT-04	9.3	1.5
VUMC-EPN-06	9.8	1.8
VUMC-EPN-05	9.5	1.8
VUMC-EPN-01	9.4	1.6
KNS-42	9.9	2
VUMC-DIPG-11	7.8	1
VUMC-DIPG-F	10	1.7
OPBG-GBM-001	9.4	1.6

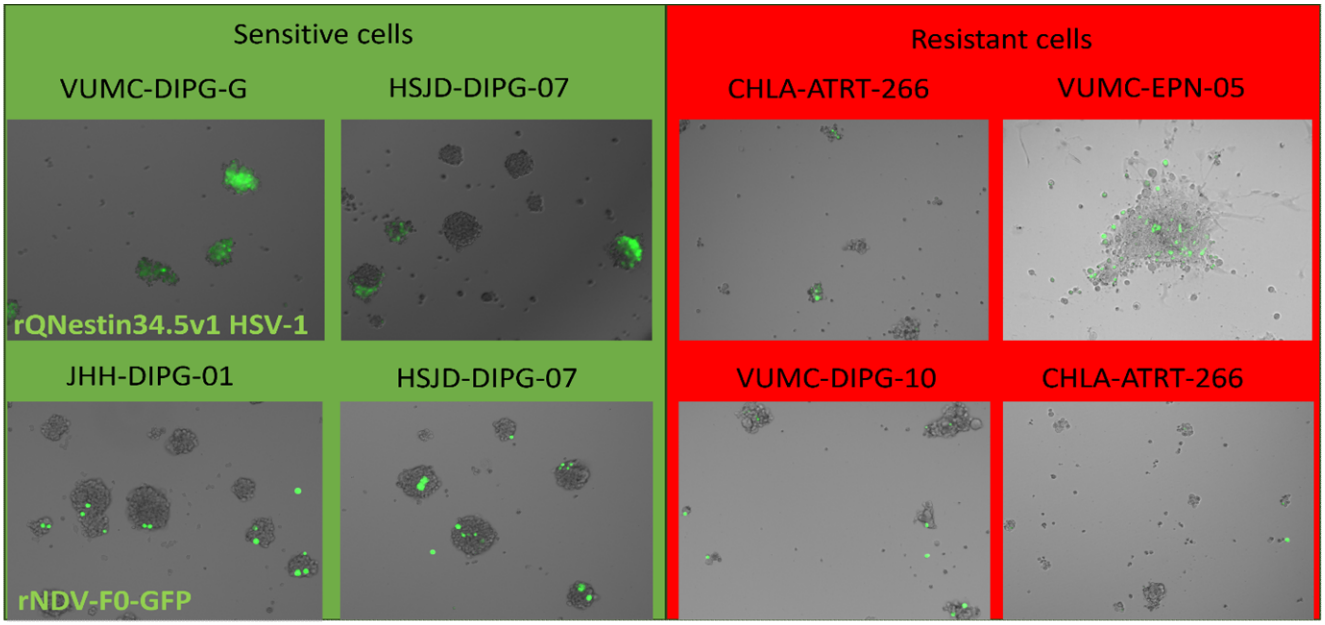

Figure S1. Immunofluorescent depiction of GFP-tagged OV activity.

Immunofluorescent images (20x) of the viral activity of rQNestin34.5v1 and rNDV-F0-GFP five days p.i. with MOI 3.3 and 1.1, respectively, of two representative sensitive PBTs (for rQNestin34.5v1: VUMC-DIPG-G and HSJD-DIPG-07, for rNDV-F0-GFP: JHH-DIPG-01 and HSJD-DIPG-07) and two representative resistant PBTs (for rQNestin34.5v1: CHLA-ATRT-266 and VUMC-EPN-05, for rNDV-F0-GFP: VUMC-DIPG-10 and CHLA-ATRT-266) .
